# Development of hydroxybenzoic-based platforms as a solution to deliver dietary antioxidants to mitochondria

**DOI:** 10.1038/s41598-017-07272-y

**Published:** 2017-07-28

**Authors:** José Teixeira, Catarina Oliveira, Ricardo Amorim, Fernando Cagide, Jorge Garrido, José A. Ribeiro, Carlos M. Pereira, António F. Silva, Paula B. Andrade, Paulo J. Oliveira, Fernanda Borges

**Affiliations:** 1 CIQUP/Department of Chemistry and Biochemistry, Faculty of Sciences, University of Porto, Porto, 4169-007 Portugal; 2 CNC – Center for Neuroscience and Cell Biology, UC-Biotech Building, Biocant Park –University of Coimbra, Cantanhede, 3060-197 Portugal; 3 Department of Chemical Engineering, School of Engineering (ISEP), Polytechnic of Porto, Porto, 4200% Portugal; 4 REQUIMTE/LAQV-Laboratory of Pharmacognosy, Department of Chemistry, Faculty of Pharmacy, University of Porto, Porto, 4050-313 Portugal

## Abstract

Oxidative stress and mitochondrial dysfunction have been associated with metabolic and age-related diseases. Thus, the prevention of mitochondrial oxidative damage is nowadays a recognized pharmacological strategy to delay disease progression. Epidemiological studies suggested an association between the consumption of polyphenol-rich diet and the prevention of different pathologies, including diseases with a mitochondrial etiology. The development of mitochondrial-targeted antioxidants based on dietary antioxidants may decrease mitochondrial oxidative damage. Herein, we report the design and synthesis of two new mitochondriotropic antioxidants based on hydroxybenzoic acids (AntiOxBENs). The results obtained showed that the novel antioxidants are accumulated inside rat liver mitochondria driven by the organelle transmembrane electric potential and prevented lipid peroxidation, exhibiting low toxicity. Some of the observed effects on mitochondrial bioenergetics resulted from an increase of proton leakage through the mitochondrial inner membrane. The new derivatives present a higher lipophilicity than the parent compounds (protocatechuic and gallic acids) and similar antioxidant and iron chelating properties. AntiOxBENs are valid mitochondriotropic antioxidant prototypes, which can be optimized and used in a next future as drug candidates to prevent or slow mitochondrial oxidative stress associated to several pathologies.

## Introduction

Polyphenols are secondary plant metabolites mostly involved in defence against oxidative stressors that are found largely in fruits, vegetables, cereals, and beverages present in human diet^1, 2^. Their daily dietary intake in the regular Western diet was estimated to be about 1 g. Epidemiological studies and associated meta-analyses suggested a strong association between the consumption of polyphenol-rich diets and the prevention of conditions such as cancer, diabetes, cardiovascular and neurodegenerative diseases^3, 4^.

Hydroxybenzoic acids (HBAs), a subclass within phenolic acids, comprises seven carbon atoms (C6-C1) linked to at least one hydroxyl group. Some HBA derivatives are currently used as additives to prevent or minimize the oxidation of nutrients and to maintain or improve the food nutritional value^5^ and as excipients in cosmetic and pharmaceutical industries due to their antioxidant properties^6^.

The antioxidant activity of HBAs has been associated with their chelating and free radical scavenging properties, namely in preventing lipid peroxidation processes^7–9^, and with their role in the inhibition of several pro-oxidant enzymes, which are involved in reactive oxygen species (ROS) production^10–12^.

The usefulness of HBAs in human therapy, alone or as adjuvants, is restricted due to bioavailability and druggability limitations^2, 13^, a problem that is mainly related with their physicochemical properties (e.g. lipophilicity) and a rapid and extensive metabolism^14^. Accordingly, different strategies have been advanced to increase HBAs lipophilicity and stability and for improving their delivery to an intracellular targets^11, 15^.

Mitochondria play a vital role in regulating energy metabolism, cytosolic calcium concentration, ROS production, and cell death pathways^16^. Excessive ROS production, if not counteracted by intrinsic defence mechanisms, can cause oxidative damage on cellular components such as lipids, proteins and nucleic acids and in turn trigger subsequent cell death by necrosis or apoptosis. Mitochondrial alterations resulting from augmented oxidative stress play a crucial role in several diseases such as cancer, stroke, heart failure, obesity and neurodegenerative disorders^17, 18^. Different approaches have been established to target mitochondria including the development of electron transport chain (ETC) inhibitors, oxidative phosphorylation (OXPHOS) uncouplers, mitochondrial Ca^2+^ modulators and mitochondriotropic antioxidants^18^. One of the most studied mitochondria-targeted antioxidants is Mitoquinone (MitoQ), which consists in an endogenous antioxidant moiety (coenzyme Q) covalently linked to a triphenylphosphonium cation (TPP) by a 10-carbon alkyl chain (dTPP), a lipophilic spacer which allows for the molecule to cross mitochondrial membranes^19^.

**Figure 1:**
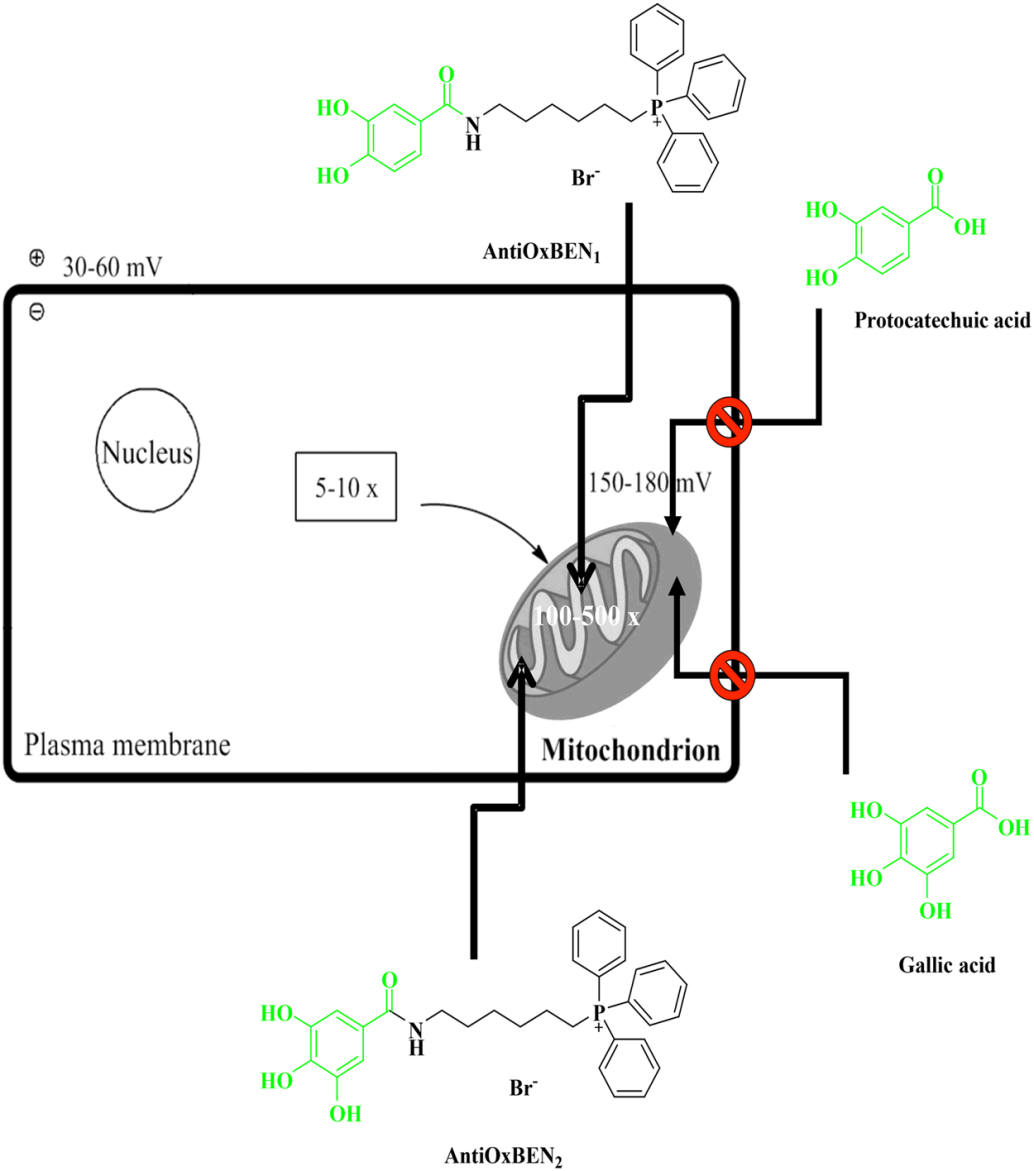
Design of mitochondriotropic antioxidants (AntiOxBEN_1_ and AntiOxBEN_2_) based on dietary scaffolds (protocatechuic and gallic acids).

As part of our long-term project related with the development of effective antioxidants based on natural models, we report here the production of novel mitochondrial-directed antioxidant based on natural dietary HBAs, namely protocatechuic (AntiOxBEN_1_) and gallic acid (AntiOxBEN_2_) (Fig. 1). Hereafter, the synthesis, antioxidant, redox and lipophilic properties and mitochondrial interactions of the new AntiOxBENs compounds are described.

## Results

### Chemistry

**Figure 2:**
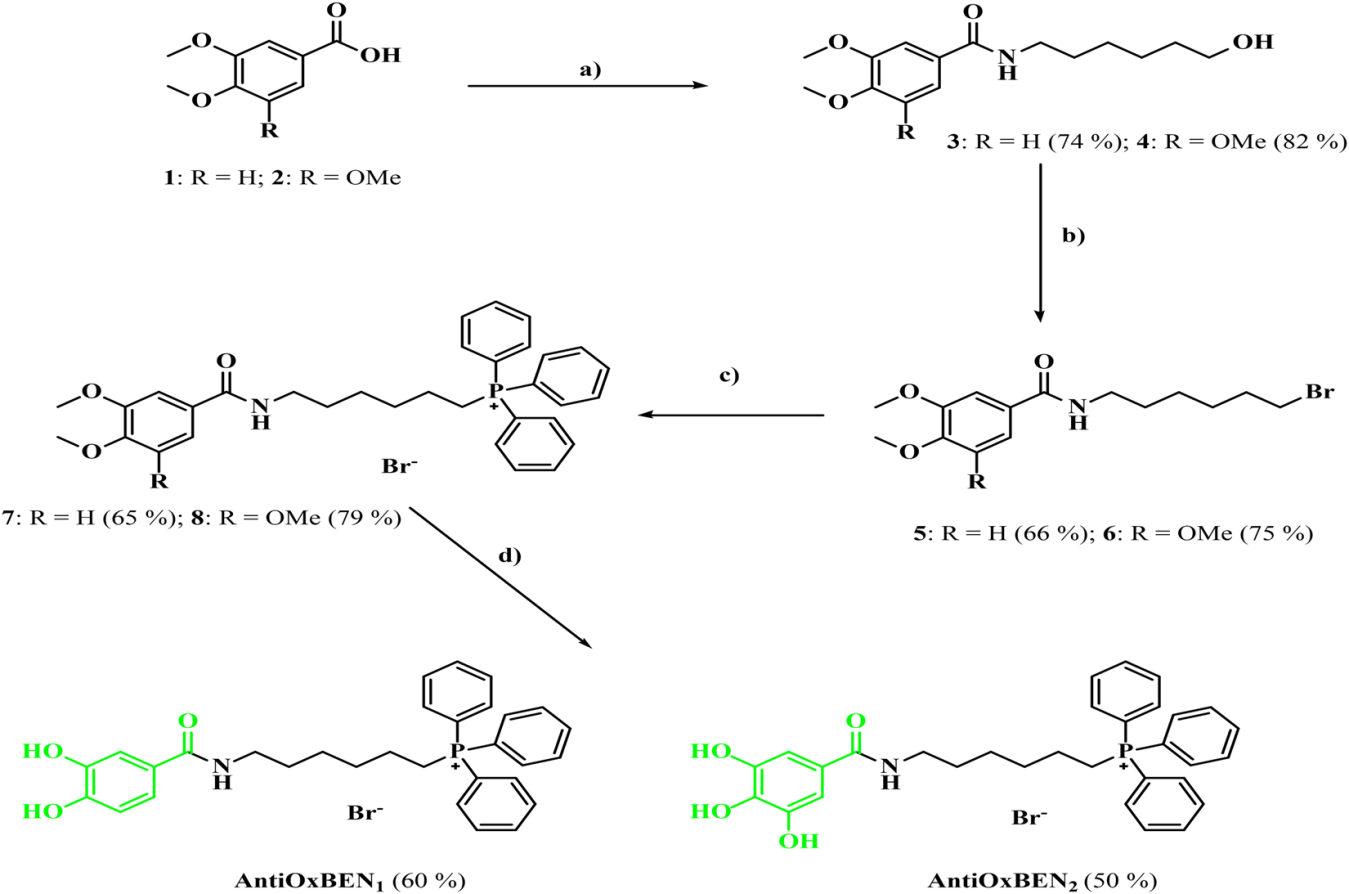
Synthetic strategy used for production of mitochondriotropic antioxidants based on protocatechuic acid (AntiOxBEN_1_) and gallic acid (AntiOxBEN_2_). Reagents and conditions: (**a**) triethylamine, ethyl chloroformate, 6-aminohexan-1-ol, dichloromethane, r.t. (12 h); (**b**) 1,2-dibromotetrachloroethane, 1,2-bis(diphenylphosphine)ethane (*diphos*), tetrahydrofuran, r.t. (24 h); (**c**) Triphenylphosphine, 120 °C (48 h); (**d**) BBr_3_, anhydrous dichloromethane, from −70 °C (10 min) to r.t. (12 h).

The mitochondriotropic antioxidants AntiOxBEN_1_ and AntiOxBEN_2_ were obtained by the four synthetic step strategy depicted in Fig. 2.

In the first step, the starting materials di (**1**) or trimethoxybenzoic (**2**) acids were linked to a bifunctionalized alkyl spacer (6-aminohexan-1-ol) by an amidation reaction using ethylchloroformate as coupling reagent. The second step reaction was aimed to convert the alcohol function (compounds **3** or **4**) into a halide, which is a good leaving group (Fig. 2). Several synthetic approaches have been used for this, namely the use of phosphorus tribromide (PBr_3_), either by classic or microwave conditions. However, the purification steps were difficult and yields were low (20–35%) in all reactions. The desired compounds were obtained in high yields (66% and 75%, for compounds **5** and **6**, respectively) by Appel-modified reaction using 1,2-bis(diphenylphosphino)ethane (*diphos*)^20^ instead of triphenylphosphine, the classic Appel nucleophile. *Diphos* was converted to readily-filtered dioxide byproducts allowing surpassing the purification process drawbacks characteristic of the Appel reaction. In a third step, the triphenylphosphonium salts (compounds **7** or **8**) were obtained via a S_N_2 reaction displaced by triphenylphosphine (PPh_3_). The synthesis of **AntiOxBEN**_**1**_ and **AntiOxBEN**_**2**_ was performed by a demethylation process using boron tribromide (BBr_3_)^21^. The compounds were identified by spectroscopic techniques: NMR (^1^H and ^13^C NMR) and MS-ESI.

### AntiOxBENs radical scavenging activity

AntiOxBENs antioxidant ranking activity hierarchy was established by *in vitro* cell-free methods often used in drug discovery processes. In total antioxidant capacity assays (TAC), such as DPPH^•^ (2,2′-diphenyl-1-picrylhydrazyl radical) and ABTS^•+^ (2,2′-azino-bis(3-ethylbenzthiazoline-6-sulfonic acid), the ability of an antioxidant to transfer a hydrogen atom, or an electron, to a stable free radical is measured by the radical absorbance decrease as a result of an *in situ* radical deactivation by an antioxidant. Compounds with higher antioxidant activity display a higher % of radical inhibition.

**Figure 3:**
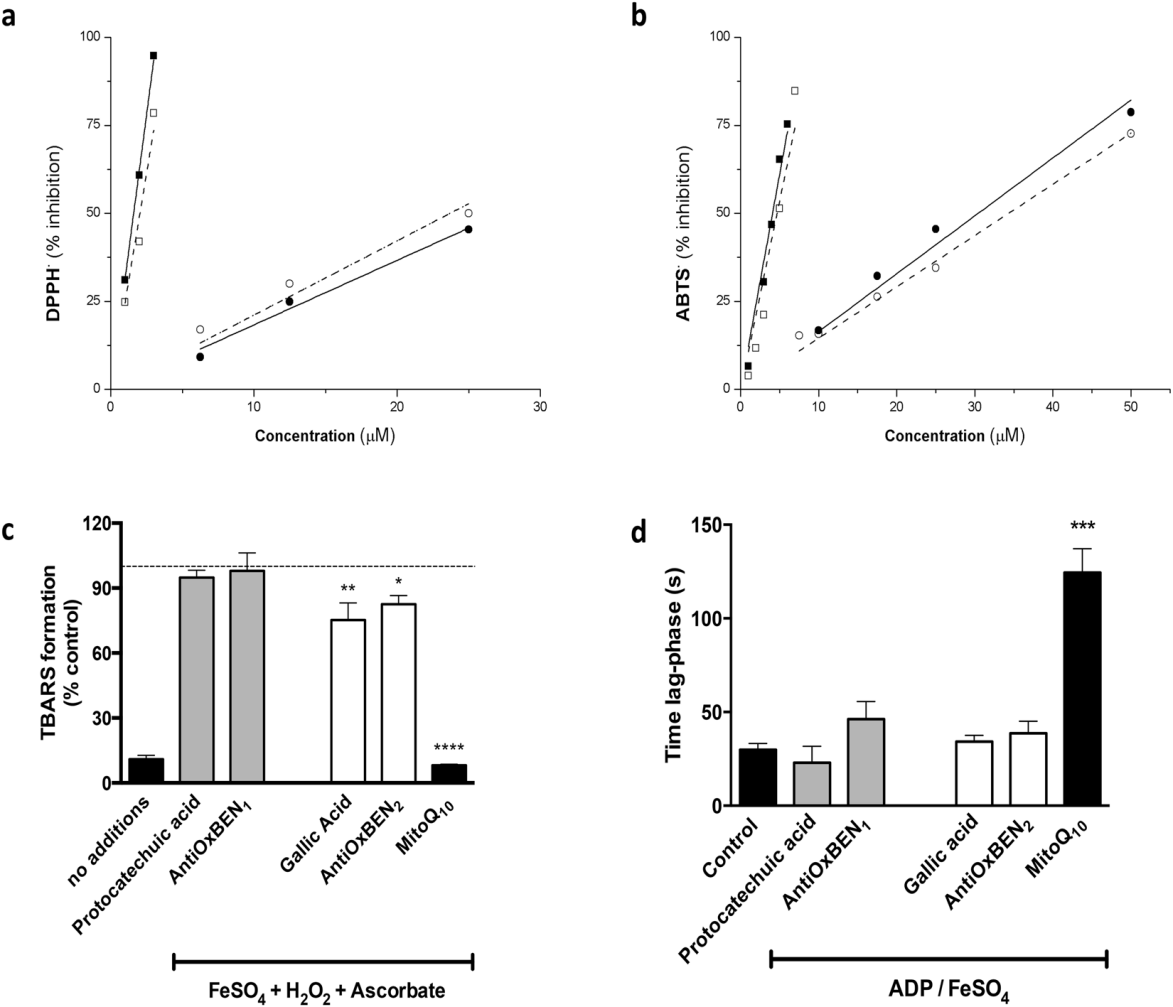
Antioxidant outline of mitochondria-targeted benzoic antioxidants. Radical scavenging activities of hydroxybenzoic acid derivatives on (**a**) DPPH and (**b**) ABTS radicals. •protocatechuic acid; ◾gallic acid; οAntioOxBEN_1_; and ◽AntioOxBEN_2_. Effect of AntiOxBENs on mitochondrial lipid peroxidation: (**c**) TBARS levels and (**d**) oxidation-derived oxygen consumption under different oxidative conditions. Data are means ± SEM from three and six independent experiments and are expressed as % of control (control = 100%) for TBARS and time lag-phase (s) for oxidation-derived oxygen consumption assays, respectively. The comparisons between control preparation vs. AntiOxBENs (5 μM) pre-incubations were performed by using one-way ANOVA. Significance was accepted with *P < 0.05, **P < 0.01, ***P < 0.0005, ****P < 0.0001.

The antioxidant data obtained in different assays showed that AntiOxBENs are effective antioxidants. For the new pyrogallol (AntiOxBEN_2_) and catechol (AntiOxBEN_1_) systems a slight decrease in antioxidant activity, when compared to their precursors (gallic or protocatechuic acids) was observed, which was probably related to the effect of the triphenylphosphonium (TPP) aliphatic side chain (Fig. 3a and b). Moreover, the pyrogallol based system (AntiOxBEN_2_) displayed a superior antioxidant activity than catechol (AntiOxBEN_1_).

### AntiOxBENs iron chelation properties

Phenolic antioxidants can operate by different mechanisms of action, namely by scavenging deleterious reactive species and/or by chelation of pro-oxidant transition metals (namely Cu and Fe). In this context, AntiOxBENs iron chelating properties were evaluated.

The iron (II) chelation capacity of AntiOxBENs was evaluated by the ferrozine assay using EDTA (ethylenediaminetetracetic acid) as reference (Figure S3). The iron chelating properties of protocatechuic and gallic acids, as well as MitoQ_10_, a classic mitochondriotropic antioxidant, were also evaluated.

As expected, EDTA chelated all the iron available in solution as it can inhibit completely the formation of the colored ferrozine-fe(II) complex. AntiOxBENs (catechol or pyrogallol-based) and hydroxybenzoic acids, in opposition to MitoQ_10_, were able to chelate ferrous iron (Table 1). The chelating properties of AntiOxBEN_1_ and AntiOxBEN_2_ seem to be to some extent affected by the introduction of the TPP cation spacer, when compared with the respective precursors. Yet, AntiOxBEN_2_ chelated more than 80% of the total iron present in solution.

**Table 1:** Properties of hydroxybenzoic acids and mitochondria-targeted benzoic antioxidants.

Compound	MW (gmol^−1^)	*E*_p_ (V)	E_tr_/V	%Fe(II) chelation	Accumulation Ratio RLM
Protocatechuic acid	154.12	0.176;0.315	—	81.1	—
AntiOxBEN_1_	578.48	0.219	0.405	65.6	2200
Gallic acid	170.12	0.120;0.562	—	95.9	—
AntiOxBEN_2_	594.48	0.115	0.495	82.6	1900

### AntiOxBENs effects on mitochondria lipid peroxidation

The antioxidant activity of HBAs has been associated with their scavenging free radical properties, namely as inhibitors of lipid peroxidation. Thus, AntiOxBENs antioxidant activity toward lipid peroxidation of RLM membranes was determined. Two different oxidative stressors, FeSO_4_/H_2_O_2_/ascorbate and ADP/FeSO_4_, and two end-points, TBARS production and oxygen-consumption, were used. MitoQ_10_ was used as reference (Fig. 3).

When measuring TBARS, gallic acid and AntiOxBEN_2_ were the most effective hydroxybenzoic acid derivatives in preventing mitochondrial lipid peroxidation, while AntiOxBEN_1_ and protocatechuic acid were not effective in preventing TBARS formation in RLM (Fig. 3c). Time-dependent oxygen consumption (Figure S2) resulting from the lipid peroxidation of RLM membranes was also monitored^22, 23^. The time lag-phase that followed ADP/Fe^2+^ addition was used to measure the AntiOxCINs efficiency (Fig. 3d). In the ADP/FeSO_4_ assay, none of AntiOxBENs efficiently prevented lipid peroxidation.

The ability of AntiOxBENs vs MitoQ to inhibit lipid peroxidation in RLM decreased in the order MitoQ ≫ AntiOxBEN_2_ ≈ gallic acid > AntiOxBEN_1_ ≈ protocatechuic acid. In general, pyrogallol-based AntiOxBEN_2_ was more effective in delaying lipid peroxidation membrane process.

### AntiOxBENs redox properties

Since antioxidants are effective by two major mechanisms, hydrogen atom transfer (HAT) and single electron transfer (SET), electrochemistry assays can provide valuable information regarding their antioxidant properties. Thus, the oxidative behaviour of AntiOxBENs and parent antioxidants (protocatechuic and gallic acids) was evaluated at physiological pH 7.4, by using differential pulse and cyclic voltammetry, using a glassy carbon working electrode.

**Figure 4:**
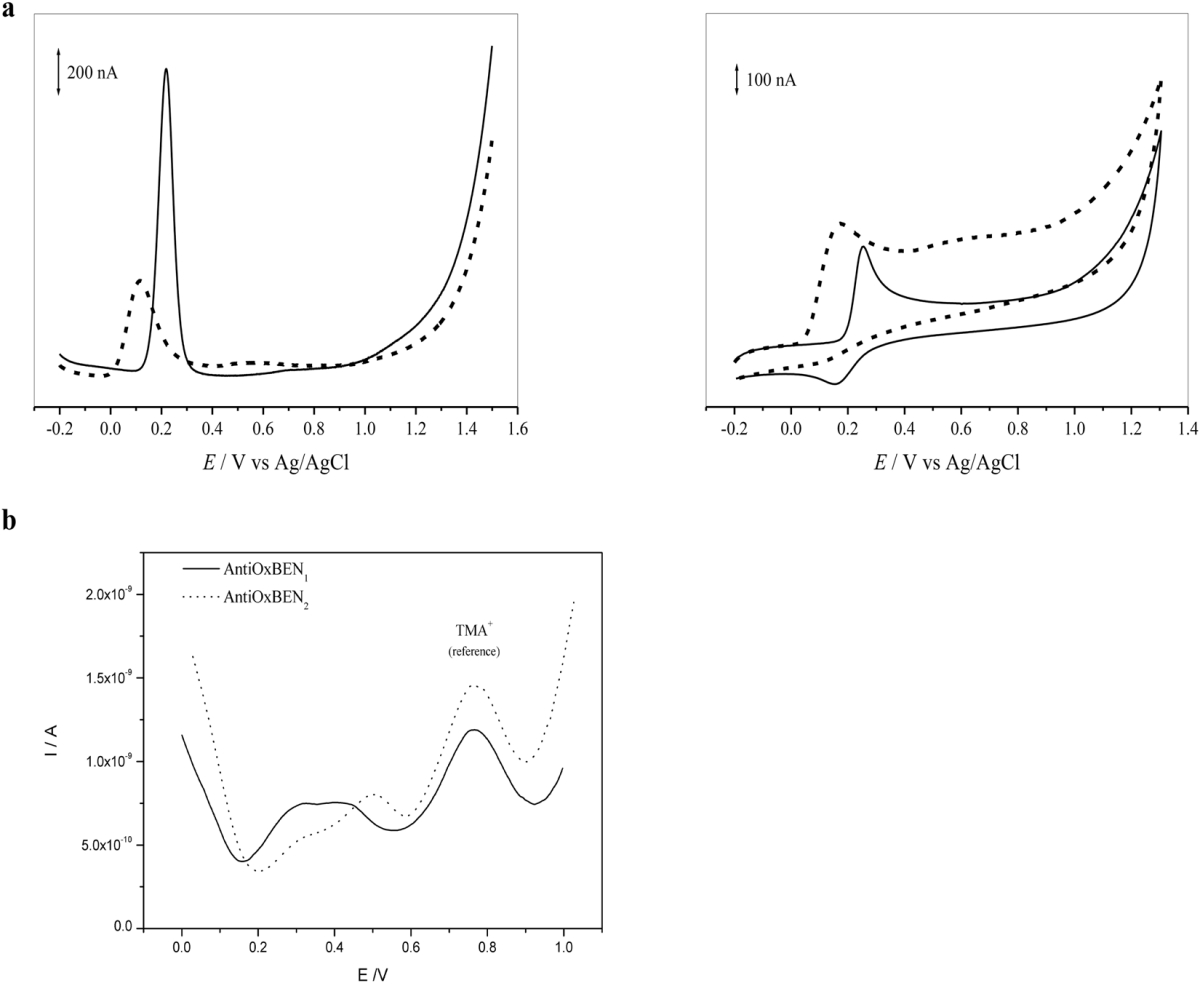
(**a**) Representative AntiOxBENs voltammograms: (upper panel) Differential pulse and (lower panel) cyclic voltammograms for a 0.1 mM solution of (▬) AntiOxBEN_1_ and (●●●) AntiOxBEN_2_ at physiological pH 7.4 supporting electrolyte. Scan rate: 5 mV s^−1^ (DPV) and 20 mV s^−1^ (CV). (**b**) Evaluation of AntiOxBENs lipophilicity in water/DCH. Differential pulse voltammograms representing the transfer of 0.1 mM of AntiOxBEN_1_ (▬) and AntiOxBEN_2_ (●●●) at the water/DCH micro-interface at physiological pH 7.4. [TMA^+^] = 0.13 mM. Scan rate: 8 mV s^−1^ (DPV).

The differential pulse voltammetric study of protocatechuic acid showed the presence of two convolved anodic peaks (resulting from electron transfer for both free and adsorbed forms) at physiological pH (Table 1). However, only one anodic wave was observed for the mitochondriotropic antioxidant AntiOxBEN_1_. The oxidation peaks observed for both compounds are related to the oxidation of the catechol group present in their structures (Fig. 4a). The occurrence of a single voltammetric wave for AntiOxBEN_1_ can be related with its lower propensity to adsorb on the electrode surface when compared to the parent acid^11^. The cyclic voltammograms obtained for both compounds shows one anodic and the corresponding cathodic peak at a scan rate of 20 mV/s, but the difference between anodic and cathodic peak potential value indicates an irreversible electron-transfer process (Fig. 4a). Similarity to protocatechuic acid and derivatives, the oxidation involves two electrons and two protons per molecule, which likely correspond to the *in situ* formation of semiquinone radicals and subsequent oxidation to ortho-quinone^11, 24^.

The differential pulse voltammetric study of gallic acid and its derivative (AntiOxBEN_2_) revealed the presence of two well-defined anodic waves at physiological pH (Table 1). The oxidation peaks are related to the oxidation of the pyrogallol unit present in their structure (Fig. 4a). Cyclic voltammetric experiments presented a single oxidation peak with no distinct reduction wave on the reverse sweep, showing that gallic acid and AntiOxBEN_2_ were irreversibly oxidized (Fig. 4a). Similar to gallic acid and its derivatives, the mechanism can occur *via* two electrons and two protons per molecule, which likely correspond to the formation of an *ortho*-quinone^25, 26^.

In summary, the redox data allow concluding that protocatechuic acid and AntiOxBEN_1_ showed redox potentials (Ep) characteristic of the presence of a catechol group (Ep = 0.257 and 0.224 V, respectively) and that for pyrogallol derivatives (gallic acid and AntiOxBEN_2_), a significant decrease in redox potentials was observed (Ep = 0.163–0.168 V) (Table 1).

### AntiOxBENs lipophilic properties

Electrochemistry at the interface between two immiscible electrolyte solutions (ITIES) is a technique often used to mimic transfer of ionic drugs through biological membranes^27, 28^. Accordingly, the AntiOxBENs lipophilic properties were evaluated using differential pulse voltammetry (DPV) at physiological pH by measuring the transfer potential (E_tr_) at which the ionic drug initially present in the aqueous phase (C = 0.32 mM) is transferred to 1,6-dichlorohexane (DCH) phase. The current charge increments observed in the voltammograms corresponded to AntiOxBENs transfer from water to the DCH phase (Fig. 4b). The AntiOxBENs transfer potentials (E_tr_) are shown in Table 1. The presence of an additional OH function in AntiOxBEN_2_ (mitochondria-targeted antioxidant based on gallic acid) increased hydrophilicity in comparison with AntiOxBEN_1_ (mitochondria-targeted antioxidant based on protocatechuic acid), which was translated in a rise of the transfer potential. As expected, due to their hydrophilicity, hydroxybenzoic acids did not permeate.

### AntiOxBENs uptake by mitochondria

AntiOxBENs mitochondrial uptake was assessed in isolated rat liver mitochondria (RLM) in response to the transmembrane electric potential (∆Ψ)^29^. The addition of complex II substrate succinate resulted in ∆Ψ generation and consequent AntiOxBENs accumulation inside mitochondria driven by the Δψ. The accumulated AntiOxBENs were then released from mitochondria since the ∆Ψ was abolished by the K^+^-ionophore valinomycin (Figure S3B). The results clearly showed a ∆Ψ-dependent uptake of AntiOxBENs. Different AntiOxBENs mitochondrial accumulation profiles have been measured (Table 1 and Figure S3C). The process was found to be dependent of their aromatic ring substitution pattern.

### AntiOxBENs effects on mitochondrial bioenergetics

AntiOxBENs and MitoQ_10_ toxicity effects on liver mitochondrial bioenergetics, namely on ΔΨ and respiration parameters, were evaluated^30^. AntiOxCINs and MitoQ_10_ were tested at antioxidant-relevant concentrations.

The mitochondrial bioenergetics data obtained for MitoQ_10_ was shown in Figure S4 and Table S1 (for details see Supporting Information S2). The results obtained have been used for comparative analysis against the test compounds here described.

**Figure 5:**
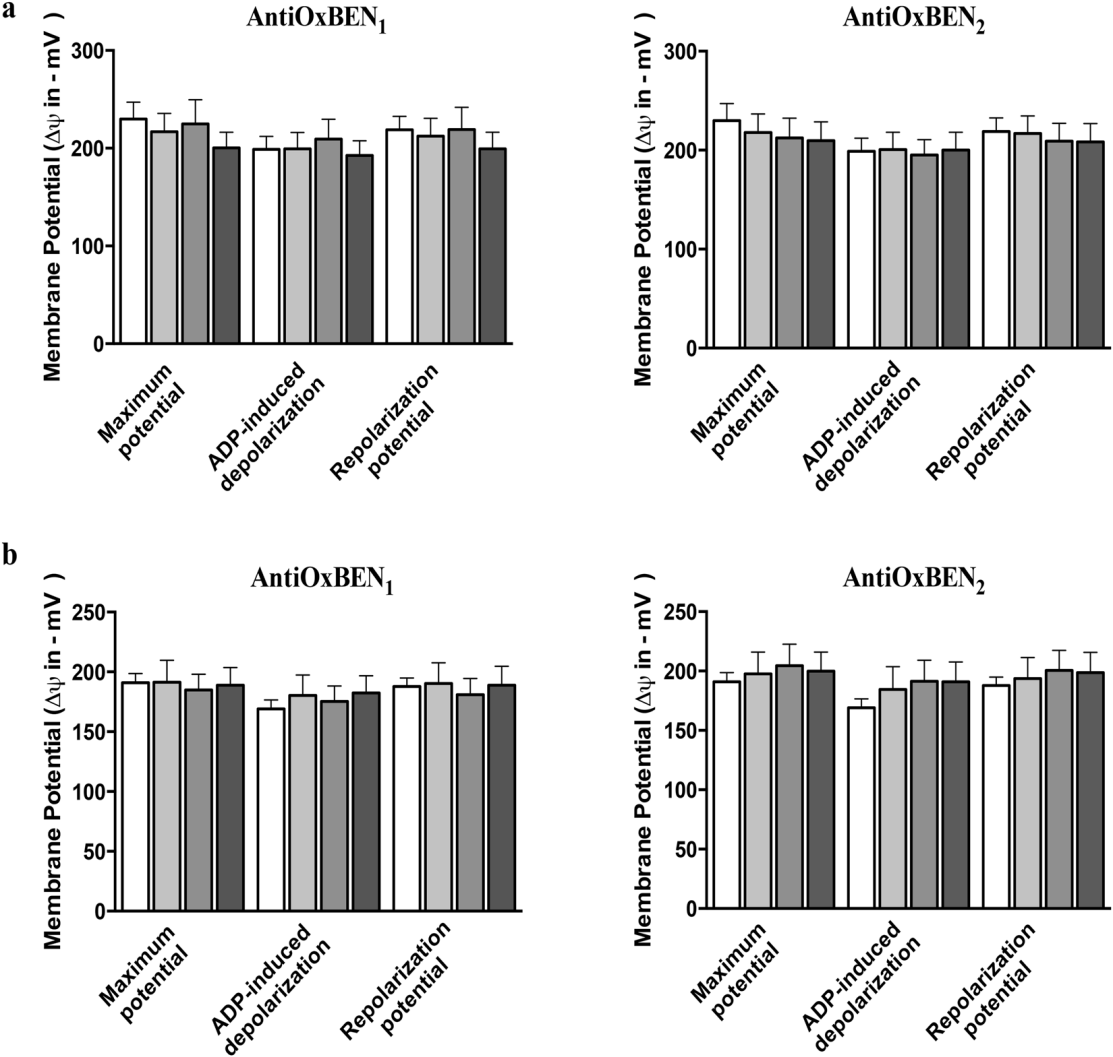
Effects of AntiOxBENs on RLM transmembrane electric potential (ΔΨ) supported by (**a**) 10 mM glutamate + 5 mM malate or (**b**) 5 mM succinate. The white bars refer to the control, while grey bars refer to the experiments where RLM were pre-incubated with AntiOxBENs (2.5 μM – light grey; 5 μM – grey; and 10 μM – dark grey). The presented results are means ± SEM of five independent experiments. The statistical significance relative to the different bioenergetics parameters was determined using Student’s two tailed t-test (*P < 0.05, **P < 0.01, ***P < 0.0005, ****P < 0.0001).

The ΔΨ represents the main component of the proton electrochemical gradient generated by mitochondrial respiration and accounts for more than 90% of the total available energy. Direct effects of AntiOxBENs on ΔΨ were evaluated using glutamate-malate (which generates NADH for complex I) (Fig. 5a) and succinate (reducing complex II) (Fig. 5b) as substrates to energize RLM isolated fractions. Mitochondria developed a ΔΨ ≈ 230 mV and ΔΨ ≈ 186 mV (negative inside) upon energization with glutamate/malate and succinate, respectively (Fig. 5). AntiOxBENs ∆Ψ alterations were dependent of the substrate used. After glutamate/malate-energization AntiOxBENs caused a slight ∆Ψ dose-dependent depolarization (10–20 mV) while promoting a slight hyperpolarization of 5–20 mV under succinate-energization. Still, it is important to note that AntiOxBENs did not significantly affect RLM ∆Ψ (Fig. 5).

**Figure 6:**
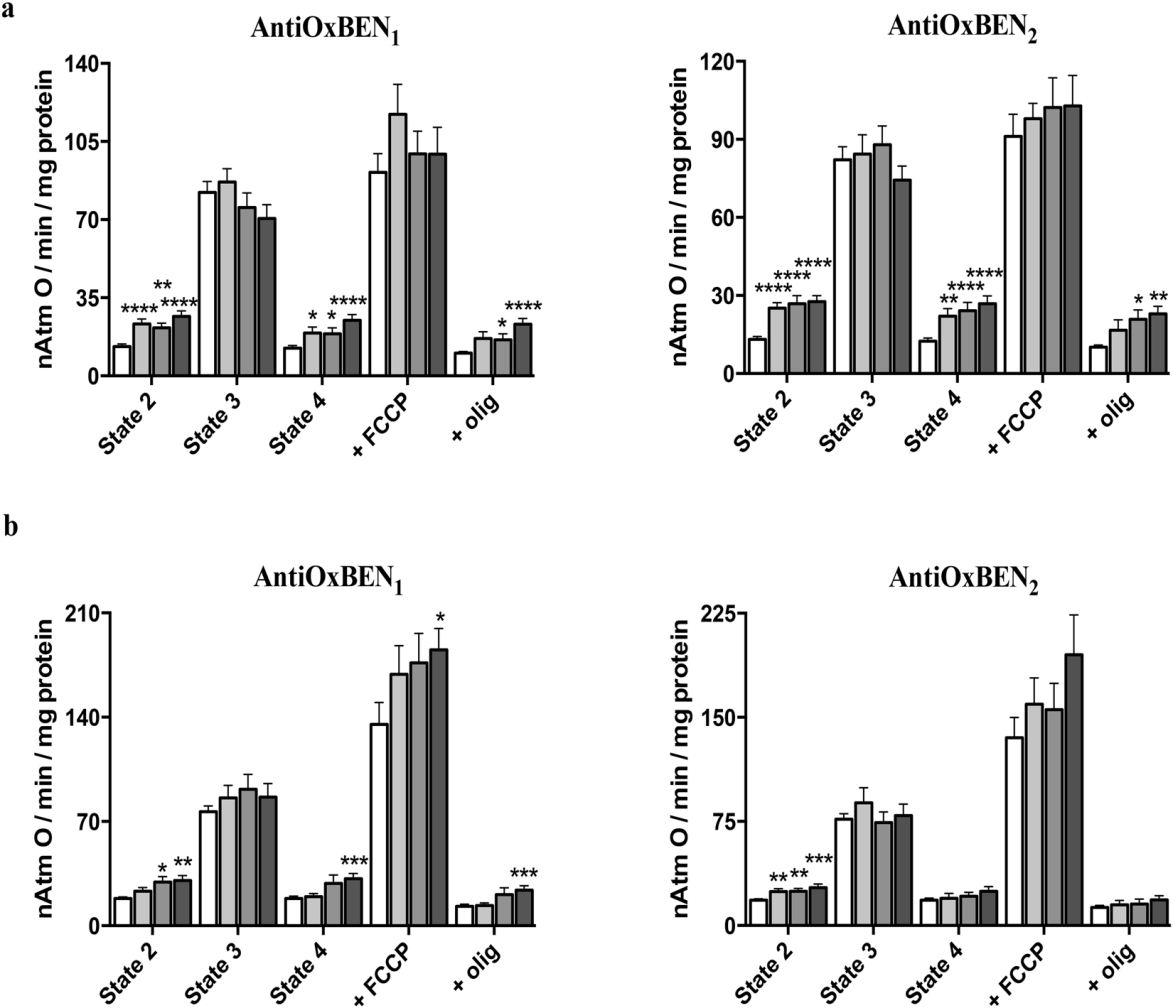
Effect of AntiOxBENs on RLM respiration supported by (**a**) 10 mM glutamate + 5 mM malate or (**b**) 5 mM succinate. The white bars refer to the control, while grey bars refer to the experiments where RLM were pre-incubated with AntiOxBENs (2.5 μM – light grey; 5 μM – grey; and 10 μM – dark grey). The presented results are means ± SEM of seven independent experiments. The statistical significance relative to the different respiratory rates/states was determined using Student’s two tailed t-test (*P < 0.05, **P < 0.01, ***P < 0.0005, ****P < 0.0001).

Mitochondrial respiration assays, including mitochondrial fitness parameters (RCR and ADP/O ratio), were evaluated to determine the mitochondrial toxicity of AntiOxBENs. The rates for state 2, state 3, state 4, oligomycin-inhibited and FCCP-uncoupled respiration are shown in Fig. 6. The mitochondrial oxidative phosphorylation coupling index, known as respiratory control ratio (RCR, state 3/state 4 respiration) was of 7.3 ± 0.6 and 4.1 ± 0.3 in the control experiments, using glutamate-malate and succinate as respiratory substrates, respectively (Table 2). ADP/O index (coupling between ATP synthesis and oxygen consumption) was 2.6 ± 0.1 and 1.5 ± 0.1 in the control experiments using complex I and complex II respiratory substrates, respectively (Table 2).

**Table 2:** Effect of AntiOxBENs on mitochondrial bioenergetics: mitochondrial respiratory control ratio (RCR) and efficiency of the phosphorylative system (ADP/O).

Mitochondrial Bioenergetics		Control	AntiOxBEN1			AntiOxBEN2		
			2.5 μM	5 μM	10 μM	2.5 μM	5 μM	10 μM
Glutamate/Malate	RCR	7.3 ± 0.6	4.9 ± 0.6*	4.4 ± 0.6**	2.6 ± 0.1***	4.3 ± 0.6**	3.9 ± 0.5**	2.9 ± 0.3****
	ADP/O	2.6 ± 0.1	2.5 ± 0.2	2.8 ± 0.4	2.1 ± 0.1**	2.3 ± 0.3	2.3 ± 0.2	2.1 ± 0.2*
Succinate	RCR	4.1 ± 0.3	4.7 ± 0.3	3.0 ± 0.3*	2.8 ± 0.3**	5.2 ± 0.9	3.8 ± 0.4	3.5 ± 0.3
	ADP/O	1.5 ± 0.1	1.7 ± 0.1	1.6 ± 0.1	1.5 ± 0.1	1.7 ± 0.1	1.7 ± 0.1	1.6 ± 0.1

Effect of AntiOxBENs on RCR and ADP/O values in energized mitochondria (5 mM glutamate/2.5 malate or 5 mM succinate). Values are means ± SEM of seven independent experiments. Statistically significant compared with control using Student’s two tailed t-test (*P < 0.05, **P < 0.01, ***P < 0.0005, ****P < 0.0001).

AntiOxBENs incubation resulted in alterations in respiratory parameters in a dose-dependent manner. AntiOxBENs increased state 2, state 4 and oligomycin-inhibited respiration at concentrations higher than 2.5 μM in a process that is mainly dependent on their lipophilicity and not on their aromatic ring pattern (catechol vs. pyrogallol) (Fig. 6). However, it must be stressed that the observed effects were more apparent by using complex I substrates. Specific respiratory alterations by using complex I substrates resulted in a significant decrease of the respiratory control ratio (RCR) (Table 2). Moreover, AntiOxBENs (10 μM) also affected the mitochondrial phosphorylative system, as assessed by alterations in the ADP/O ratio (Table 2). At the same concentration, AntiOxBEN_1_ markedly increase FCCP-uncoupled respiration upon succinate-energization (Fig. 6).

Still, independently of their mechanism, AntiOxBENs RLM toxicity was only detected at higher concentrations than those found to exert antioxidant effect.

### AntiOxBENs cytotoxicity outline on rat cardiomyoblasts, human neonatal dermal fibroblasts and human hepatoma cells

**Figure 7:**
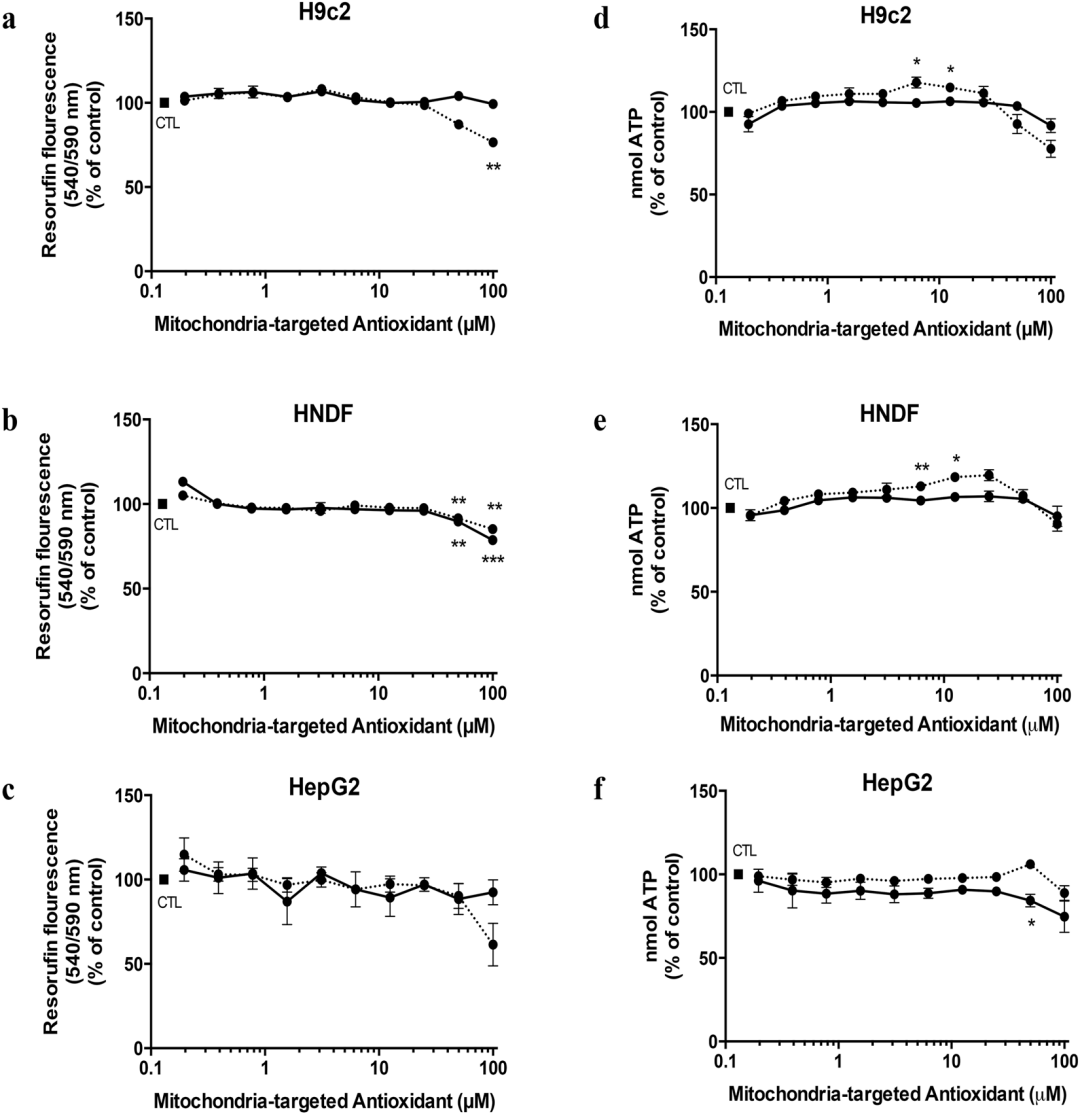
Cytotoxicity profile of AntiOxBEN_1_ (−) and AntiOxBEN_2_ (−) on (**a**) rat embryonic cardiomyoblasts (H9c2), (**b**) human neonatal dermal fibroblasts (HNDF) and (**c**) human hepatocellular carcinoma (HepG2) cells. AntiOxBEN_1_ (−) and AntiOxBEN_2_ (−) cytotoxicity determined by changes in intracellular ATP levels on (**d**) rat embryonic cardiomyoblasts (H9c2), (**e**) human neonatal dermal fibroblasts (HNDF) and (**f**) human hepatocellular carcinoma (HepG2) cells. Data are means ± SEM of four independent experiments and the results are expressed as percentage of control (control = 100%), which represents the cell density without any treatment in the respective time point. Statistically significant compared with control group using one-way ANOVA. Significance was accepted with *P < 0.05, **P < 0.01, ***P < 0.0005, ****P < 0.0001.

AntiOxBENs cytotoxicity was evaluated in cell-based assay systems, often used in the preclinical safety assessment of drug candidates. Accordingly, H9c2 (rat embryonic cardiomyoblasts), HNDF (human neonatal dermal fibroblasts) and HepG2 (human hepatocellular carcinoma) cells were used and cytotoxicity evaluated by using the resazurin reduction fluorimetric assay (Fig. 7). From the data obtained, AntiOxBEN_1_ (catechol moiety) and AntiOxBEN_2_ (pyrogallol moiety) exhibited similar toxicity profile toward H9c2 (Fig. 7a), HNDF (Fig. 7b), HepG2 (Fig. 7c). In general, both AntiOxBENs exhibited low toxicity toward different cell lines, being a decrease on metabolic activity only observed for the highest concentration used (100 μM). AntiOxBENs ranking toxicity hierarchy on different cell lines was established: HepG2 < H9c2 < HNDF.

### AntiOxBENs do not decrease ATP intracellular content

H9c2 (Fig. 7d), HNDF (Fig. 7e) and HepG2 (Fig. 7f) cells were treated with AntiOxBENs for 48 hours prior the measurement of ATP levels. From the data obtained, AntiOxBEN_1_ (catechol moiety) and AntiOxBEN_2_ (pyrogallol moiety) clearly did not decrease ATP intracellular levels in different cells for all tested concentrations. Interestingly, AntiOxBEN_1_ (6.25 μM and 12.5 μM), but not AntiOxBEN_2_ significantly increased ATP levels in H9c2 and HNDF cells. In general, both AntiOxBENs exhibited low toxicity towards different cell lines.

### AntiOxBENs prevented oxidative stress-induced cell death

**Figure 8:**
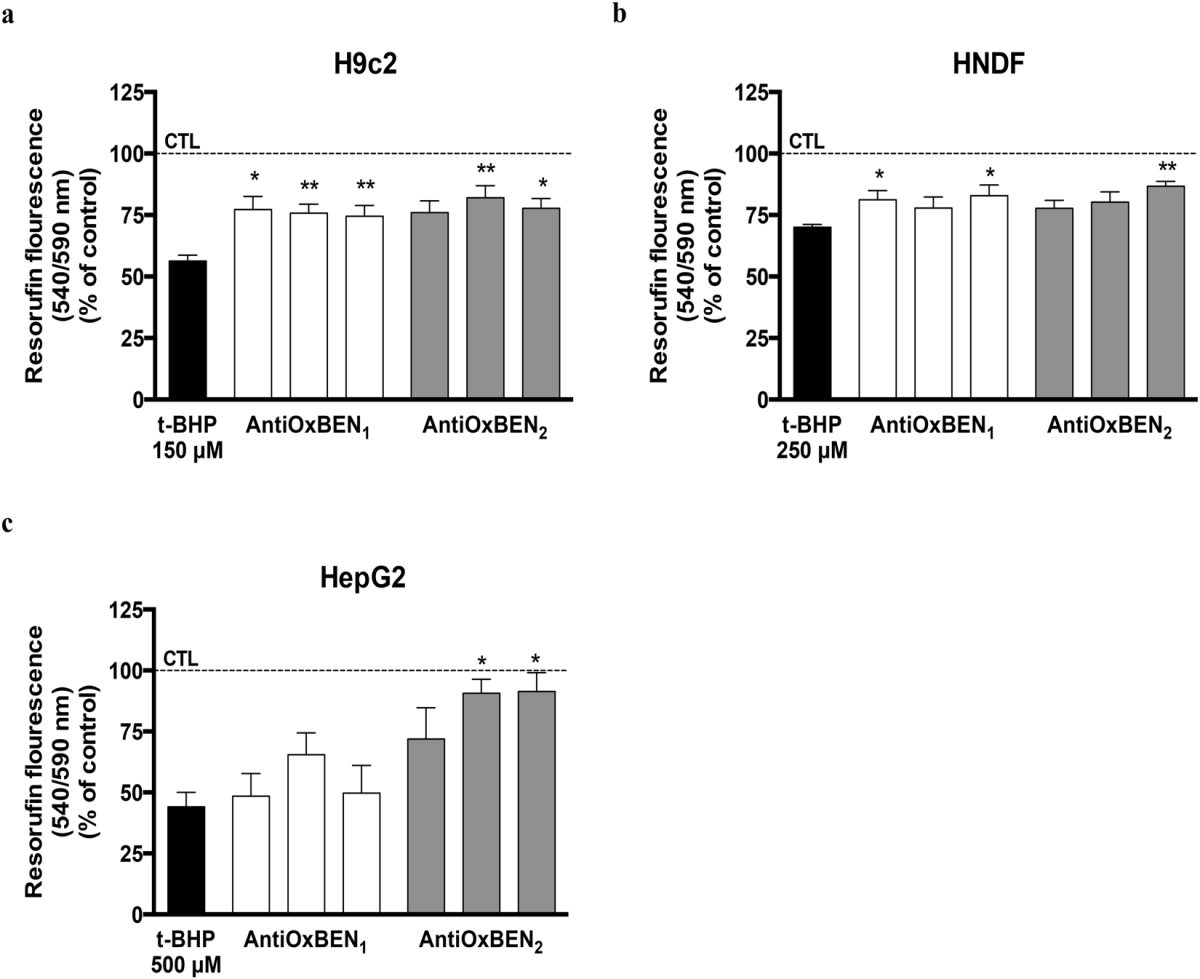
Antioxidant cytoprotective effects of AntiOxBEN_1_ and AntiOxBEN_2_ on (**a**) rat embryonic cardiomyoblasts (H9c2), (**b**) human neonatal dermal fibroblasts (HNDF) and (**c**) human hepatocellular carcinoma (HepG2) cells against t-BHP-induced metabolic activity decrease. Each compound has three bars, which corresponds to the different concentrations used (from left to right, 25, 50, 100 µM).The comparisons were performed by using one-way ANOVA between the control (t-BHP) vs. preparation where AntiOxBENs were pre-incubated. Data are means ± SEM of four independent experiments and the results are expressed as percentage of control (control = 100%), which represents the cell density without any treatment in the respective time point. Significance was accepted with *P < 0.05, **P < 0.01, ***P < 0.0005, ****P < 0.0001.

AntiOxBENs cytoprotective effects were also evaluated in cells incubated with an oxidative stressor. H9c2 (Fig. 8a), HNDF (Fig. 8b) and HepG2 (Fig. 8c) cells were exposed to oxidative stress by the addition of 150 μM, 250 μM and 500 μM of *tert*-butyl hydroperoxide (t-BHP), respectively. A decrease on metabolic activity of about 44%, 30% and 60%, respectively, was observed.

Pre-treating cells with AntiOxBENs for 24 hours before the oxidative stimulus significantly prevented t-BHP-induced cytotoxicity in both H9c2 (Fig. 8a) and HNDF (Fig. 8b) cells. AntiOxBEN_2_ exhibited a better antioxidant profile since pre-treating HepG2 (Fig. 8c) cells with AntiOxBEN_1_ for 24 hours failed to show any protective effect on t-BHP-induced cytotoxicity.

## Discussion

Mitochondria play a vital role in regulating energy metabolism, cytosolic calcium concentration, ROS production, and cell death pathways^16^. Targeting mitochondria with organelle-specific molecules is an useful therapeutic strategy for the prevention and/or treatment of oxidative stress-related diseases. In the last decade, some lipophilic cations have been developed to deliver bioactive molecules to mitochondria with the purpose of controlling or blocking mitochondrial oxidative damage^18^. Within this strategy, molecules that are poorly accumulated by mitochondria can be targeted and their concentration inside the organelle increased. MitoQ_10_^19^, based on the endogenous antioxidant coenzyme Q, and SkQ1^31^, based on the plant cofactor plastoquinone, are the most well-known mitochondria-targeted antioxidants. MitoQ_10_ has been tested under clinical trials for hepatitis C with positive outcomes^32^ while SkQ1 showed significant benefits in a human clinical trial for dry eye condition^33^.

Dietary polyphenols have been epidemiologically linked to a reduction of oxidative stress-related diseases in populations that consume high amounts of fruits or vegetables^1, 4^. In fact, it has been suggested that the administration of exogenous antioxidants can be beneficial to decrease cell injury, given that they not only compensate the insufficiency of endogenous defence systems but also improve the overall antioxidant response. Phenolic acids are secondary metabolites widely present in plants, cereals, fruits and coffee have been associated with dietary health benefits, mainly due to their antioxidant properties^1, 8^. In particular, protocatechuic and gallic acids (Fig. 1), a naturally occurring hydroxybenzoic acids (HBAs) comprising seven carbon atoms (C6-C1) connected to at least one hydroxyl group, are currently used as antioxidants in cosmetic and pharmaceutical industries and to maintain food’s nutritional value.

Our rational was the development of mitochondriotropic antioxidants based on dietary antioxidants to target and deliver HBAs to mitochondria as a strategy to overcome the bioavailability and druggability drawbacks described for polyphenols^2^, in order to increase their use in human therapy, as a drug or adjuvant. Therefore, two new mitochondriotropic hydroxybenzoic-based antioxidants were successfully designed and synthesized: one based on protocatechuic acid (AntiOxBEN_1_) and another one based on gallic acid (AntiOxBEN_2_) (Fig. 2). The new compounds present a catechol or a pyrogallol system linked by an amide bridge to a lipophilic spacer (a linear alkyl carbon chain) attached to a TPP moiety.

AntiOxBENs antioxidant profile, namely radical scavenging, as well as redox and iron chelation properties, was initially assessed to inspect whether the properties of their precursors were preserved. The data showed that AntiOxBEN_2_ and gallic acid displayed a superior antioxidant activity than AntiOxBEN_1_ and protocatechuic acid^11, 34^. AntiOxBEN_2_ and AntiOxBEN_1_ have a comparable radical scavenging activity to their precursors (Fig. 3a and b), showing that the introduction of the TPP spacer did not reduce their antioxidant activity.

AntiOxBENs, as well as HBAs, were shown to be mild RLM lipid peroxidation inhibitors (Fig. 3c). Gallic acid and AntiOxBEN_2_, in FeSO_4_/H_2_O_2_/ascorbate assay, were the most effective in preventing mitochondria lipid peroxidation^35, 36^. AntiOxBENs antiperoxidative activity ranked differently according the method used, most likely because of the different inducing agents used and their iron-chelating in the above-properties (Fig. 3c and d)^37^. Thus, it is possible that AntiOxBENs can operate by an indirect mechanism and contribute to inhibit iron-mediated ROS generation through their iron-chelation properties.

From the redox studies performed at physiological pH 7.4, it was concluded that AntiOxBENs and hydroxybenzoic acids oxidation potentials correlated well with the radical scavenging activity data (Fig. 4a): the lower oxidation potential corresponded to a higher antioxidant activity. AntiOxBEN_2_ and gallic acid displayed lower redox potentials than AntiOxBEN_1_ and protocatechuic acid. Moreover, as the introduction of a TPP cation side chain does not have a noteworthy influence on AntiOxBENs redox potentials, it can be concluded that the structural modifications performed resulted in modest or even no effect on the electron density of the catechol or pyrogallol ring. The decrease in the oxidation potential appears to be due to the existence of an additional phenolic group in gallic acid and its derivatives (pyrogallol unit). The extra hydroxyl group promotes the stabilization of the radical intermediate produced by oxidation, which was translated into a substantial decrease of the redox potential obtained. Overall, the results reinforced the assumption that the number of hydroxyl substituents present on the benzoic aromatic ring is directly related with the antioxidant and electrochemical properties.

Hydroxybenzoic acids and AntiOxBENs were able to chelate iron efficiently being those presenting a pyrogallol moiety more effective in this regard (Table 1)^7^. The chelating properties of AntiOxBEN_1_ and AntiOxBEN_2_ seemed to have been affected by the introduction of TPP cation spacer, when compared with respective precursors to some extent. This AntiOxBENs property can be of utmost importance as iron is a redox active metal, which in its free form can play a role in Fenton or Haber-Weiss reactions and the generation of hydroxyl radicals, recognized as a pathological oxidative damage primers^38^.

AntiOxBENs lipophilicity was measured by ITIES (Fig. 4b), a technique often used to mimic transfer of ionic drugs through biological membranes. Within this technique, a higher lipophilic character is translated by a less positive transfer potential^27, 28^. As expected, protocatechuic and gallic acids were not able to be transferred (Table 1). The introduction of a spacer linked to a TPP cation led to a significant improvement of HBAs lipophilicity. As AntiOxBENs have the same spacer length, it can be concluded that the introduction of an additional OH function in AntiOxBEN_2_ led to an increment of hydrophilicity (Table 1).

Next, the AntiOxBENs functional mitochondrial profile was evaluated. AntiOxBENs accumulated inside mitochondria driven by the ΔΨ, achieving intramitochondrial millimolar concentrations (Figure S3). The results clearly showed a ∆Ψ-dependent uptake: as AntiOxBEN_2_ (pyrogallol system) is less lipophilic than AntiOxBEN_1_ (catechol system), its accumulation within the mitochondrial matrix was less efficient. Still, AntiOxBENs presented an accumulation ratio comparable to that of MitoQ_10_^39^.

Mitochondrial fractions are currently used to measure the direct effect(s) of a drug on the mitochondrial bioenergetics apparatus. AntiOxBENs did not show significant protonoforetic effect as a negligible effect on ΔΨ was found (Fig. 5). Moreover, results suggested that the observed effects can probably result from a membrane permeabilization effect or a proton shuttling activity (Fig. 6). This effect may lead to stimulation of non-phosphorylation respiration and to a small ΔΨ depolarization^40, 41^ (Table 2). AntiOxBENs mitochondrial toxicity observed at higher concentrations may be associated with the lipophilicity of the spacer and/or the presence of a TPP moiety and has little, if any, relation with their (catechol vs pyrogallol)^19^. Although the presence of the TPP cation and a lipophilic spacer is essential for an efficient mitochondrial accumulation, a suitable lipophilic balance must be attained to circumvent toxicity of mitochondriotropic antioxidants. For example, although MitoQ_10_ effectively inhibited lipid peroxidation at 5 μM, it caused toxicity on the mitochondrial bioenergetic apparatus at a lower concentration (2.5 μM) under our experimental conditions (Figure S4 and Table S1). It must be stressed that AntiOxBENs RLM toxicity was measured at higher concentrations than the ones needed to exert antioxidant effect, independently of their mechanism.

A similar cytotoxic profile of both AntiOxBENs was measured on different cell models (H9c2, HNDF and HepG2) (Fig. 7). Mitochondria-targeted antioxidants containing the TPP^+^ moiety can freely pass through cellular phospholipid bilayers, with the extent of anchoring being mainly dependent upon their hydrophobicity. AntiOxBEN_1_ accumulated approximately at the same extension as AntiOxBEN_2_, so it is somehow expected that they exhibited similar cytotoxicity. Still, AntiOxBENs have a safety margin towards H9c2, HNDF and HepG2 cells. Concurrently, a new mitochondriotropic antioxidant based on gallic acid was developed by Jara *et al*.^15^ and by Cortes *et al*.^42^ aiming to disrupt mitochondrial functioning in tumor cells by a mechanism similar to the one proposed for gallic acid ester derivatives, which was related with their aromatic ring substitution pattern^15^. We showed in this work that is possible to target HBA amide derivatives to mitochondria without significantly affecting mitochondrial bioenergetics (Figs 5 and 6) and without negatively affecting ATP levels in different cell models (Fig. 7). From the data obtained, we suggest that the linker (ester vs amide) may also play a role on HBA derivatives induced-toxicity. HBA ester derivative mitochondriotropic agents are toxic and can be easily hydrolysed by esterases limiting the administration route and biological usefulness. Esterification of carboxylic group can potentiate HBAs cytotoxicity while peptide-like bond, present in AntiOxBENs, potentiate antioxidant activity. Additionally, the novel antioxidants AntiOxBEN_1_ and AntiOxBEN_2_ significantly prevented oxidative stress-induced cytotoxicity in H9c2, HNDF and HepG2 cells (Fig. 8), being AntiOxBEN_2_ the most efficient antioxidant, which is in agreement with the data from TAC, redox and RLM assays. The peptide-like bond present in AntiOxBENs make this mitochondriotropic antioxidants less toxic and more stable on biological systems. Moreover, being specifically targeted to mitochondria, they are effective at lower concentrations when compared to parental compounds^6, 43^.

Two mitochondria-targeted benzoic antioxidants (AntiOxBENs) with improved mitochondriotropic properties have been successfully designed and synthesized. AntiOxBENs had a higher lipophilicity than the parent compounds (protocatechuic and gallic acids) and similar antioxidant and iron chelating properties. AntiOxBENs are accumulated inside RLM driven by the mitochondrial membrane potential and prevented lipid peroxidation, exhibiting a low toxicity. Most of the observed effects resulted from an increase of proton leakage through the mitochondrial inner membrane. Most important, AntiOxBENs showed low mitochondrial toxicity at concentrations required for the antioxidant effect. The physicochemical properties of the dietary antioxidants protocatechuic and gallic acids have been successfully modulated and the druggability of the new derivatives demonstrated.

Facing the potent antioxidant capacity and the iron-chelating property of AntiOxBENs we predict that the innovative compounds here described may lead, after a drug discovery optimization program, to a drug candidate that can be applied to mitigate the effects of mitochondrial iron overload and/or reduce mitochondrial iron stores in oxidative stress related diseases and conditions.

## Materials and Methods

### Chemistry

#### General

3,4-Dimethoxybenzoic and 3,4,5-trimethoxybenzoic acids and all the other reagents were purchased from Sigma-Aldrich (Barcelona, Spain) and used without additional purification. The solvents were *pro analysis* grade and were acquired from Panreac (Lisbon, Portugal) and Sigma-Aldrich (Barcelona, Spain). Thin-layer chromatography (TLC) was carried out on pre-coated silica gel 60 F_254_ (Merck) with layer thickness of 0.2 mm. The spots were visualized under UV detection (254 and 366 nm) and/or an aqueous solution of ferric chloride. Flash column chromatography was performed using silica gel 60 (0.040–0.063 mm) (Carlo Erba Reactifs – SDS, France) or unmodified cellulose MN 2100 (Macherey Nagel, UK). Following the workup, the organic phases were dried over anhydrous sodium sulfate and solvents were evaporated under reduced pressure in a Büchi Rotavapor. ^1^H and ^13^C NMR spectra were acquired at room temperature and recorded on a Bruker Avance III operating at 400 and 100 MHz, respectively. Chemical shifts are expressed in δ (ppm) values relative to tetramethylsilane (TMS) as internal reference and coupling constants (J) are given in Hz. Assignments were also made from DEPT (distortionless enhancement by polarization transfer) (underlined values). Mass spectra (MS) were recorded on a Varian 320-MS (EI) or Bruker Microtof (ESI) apparatus and referred in m/z (% relative) of important fragments.

### Synthesis of AntiOxBENs

#### General synthetic procedure for obtention of hydroxyhexylbenzamides (3 and 4)

3,4-Dimethoxybenzoic acid (**1**), or 3,4,5-trimethoxybenzoic acid (**2**) (1 mmol), was dissolved in dichloromethane (40 mL) and triethylamine (2 mmol) was added. Ethylchloroformate (2 mmol) was added dropwise to the stirred solution and kept in an ice bath. After stirring 2 h at room temperature, the mixture was cooled again and 6-aminohexan-1-ol (2 mmol) was added. The reaction was stirred during 10 h at room temperature. The mixture was extracted with dichloromethane (3 × 20 mL) and the organic phases were combined, washed with water, NaHCO_3_ 5% (20 mL) and HCl 1 M (20 mL). The combined organic phases were dried and the solvent was evaporated. The product obtained was purified by silica gel flash chromatography using ethyl acetate as eluting system. The fractions containing the intended compound were collected and the solvent evaporated to obtain an amorphous solid. The reaction was followed by TLC (silica gel, ethyl acetate). The procedure was adapted from the literature^44^.

*N-(6-hydroxyhexyl)-3*,*4-dimethoxybenzamide* (**3**) Yield: 74%; TLC (EtOAc): Rf = 0.22; ^1^H NMR (400 MHz, CDCl_3_): δ 7.43 (*d*, J = 2.0 Hz, 1H, H(2)), 7.29 (*dd*, J = 8.4 Hz, J = 2.0 Hz, 1H, H(6)), 6.85 (*d*, J = 8.4 Hz, 1H, H(5)), 6.38 (*t*, J = 5.2 Hz, 1H, CONH), 3.91 (*s*, 6H, 2 × OCH_3_), 3.63 (*t*, J = 6.5 Hz, 2H, CH_2_OH), 3.45–3.40 (*m*, 2H, NCH_2_), 1.99 (*s*, 1H, OH), 1.63–1.55 (*m*, 4H, NCH_2_CH_2_(CH_2_)_2_CH_2_), 1.41–1.39 (*m*, 4H, (CH_2_)_2_(CH_2_)_2_OH); ^13^C NMR (100 MHz, CDCl_3_): δ 167.3 (CONH), 151.7 (C(4)), 149.0 (C(3)), 127.5 (C(1)), 119.4 (C(6)), 110.7 (C(2)), 110.4 (C(5)), 62.7 (CH_2_OH), 56.1 (2 × OCH_3_), 40.0 (NCH_2_), 32.6 (CH_2_CH_2_OH), 29.8 (NCH_2_ CH_2_), 26.7 (N(CH_2_)_2_ CH_2_), 25.4 (CH_2_(CH_2_)_2_OH); EI-MS m/z (%): 281 (M^+^), 208 (16), 195 (21), 194 (100), 180 (16), 165 (75), 164 (55), 121 (15).

*N-(6-hydroxyhexyl)-3*,*4*,*5-trimethoxybenzamide* (**4**) Yield: 82%; TLC (EtOAc): Rf = 0.27; ^1^H NMR (400 MHz, CDCl_3_): δ 7.00 (*s*, 2H, H(2) and H(6)), 6.28 (*t*, J = 5.1 Hz, 1H, CONH), 3.89 (*s*, 6H, 2 × OCH_3_), 3.87 (*s*, 3H, OCH_3_), 3.64 (*t*, J = 6.4 Hz, 2 H, CH_2_OH), 3.46–3.41 (*m*, 2H, NCH_2_), 1.81 (*s*, 1H, OH), 1.66–1.54 (*m*, 4H, NCH_2_CH_2_(CH_2_)_2_CH_2_), 1.43–1.40 (*m*, 4H, (CH_2_)_2_(CH_2_)_2_OH); ^13^C NMR (100 MHz, CDCl_3_): δ 167.5 (CONH), 153.3 (C(3) and C(5)), 140.9 (C(4)), 130.4 (C(1)), 104.5 (C(2) and C(6)), 62.8 (CH_2_OH), 61.0 (OCH_3_), 56.4 (2 × OCH_3_), 40.1 (NCH_2_), 32.6 (CH_2_CH_2_OH), 29.8 (NCH_2_ CH_2_), 26.7 (NCH_2_CH_2_ CH_2_), 25.4 (CH_2_(CH_2_)_2_OH); EI-MS m/z (%): 312 (M^+^), 225 (38), 224 (34), 211 (59), 196 (49), 195 (100).

#### General synthetic procedure for obtention of bromohexylbenzamides (5 and 6)

Hydroxyhexylbenzamide **3**, or **4**, (1 mmol) and 1,2-dibromotetrachloroethane (1 mmol) was dissolved in THF (20 mL). After adding 1,2-bis(diphenylphosphine)ethane (*diphos*) (0.5 mmol) the reaction was stirred at room temperature for 20 hours. Then, the reaction mixture was filtered through a Celite pad. After evaporation the oil residue obtained was purified by silica gel flash chromatography using ethyl acetate as eluting system. The fractions containing the intended compound were collected, the solvent evaporated and the solid was recrystallized from *n*-hexane. An amorphous solid was obtained. The reaction was followed by TLC (silica gel, ethyl acetate). The procedure was adapted from the literature^20^.

*N-(6-bromohexyl)-3*,*4-dimethoxybenzamide* (**5**) Yield: 66%; TLC (EtOAc): Rf = 0.67; ^1^H NMR (400 MHz, CDCl_3_): δ 7.43 (*d*, J = 2.0 Hz, 1H, H(2)), 7.27 (*dd*, J = 8.4 Hz, J = 2.0 Hz, 1H, H(6)), 6.85 (*d*, J = 8.4 Hz, 1H, H(5)), 6.25 (*t*, J = 5.4 Hz, 1H, CONH), 3.92 (*s*, 6 H, 2 × OCH_3_), 3.46–3.39 (*m*, 4H, NCH_2_(CH_2_)_4_CH_2_Br), 1.90–1.83 (*m*, 2H, CH_2_CH_2_Br), 1.67–1.59 (*m*, 2 H, NCH_2_CH_2_), 1.53–1.38 (*m*, 4H, (CH_2_)_2_(CH_2_)_2_Br); ^13^C NMR (100 MHz, CDCl_3_): δ 167.2 (CONH), 151.7 (C(4)), 149.1 (C(3)), 127.5 (C(1)), 119.2 (C(6)), 110.7 (C(2)), 110.3 (C(5)), 56.1 (OCH_3_ × 2), 40.0 (NCH_2_), 33.9 (CH_2_Br), 32.7 (CH_2_CH_2_Br), 29.7 (NCH_2_ CH_2_), 28.0 (CH_2_(CH_2_)_2_Br), 26.2 (NCH_2_CH_2_ CH_2_); EI-MS m/z (%): 345 (M^+^), 343 (24), 264 (36), 195 (34), 194 (19), 181 (40), 166 (24), 165 (100).

*N-(6-bromohexyl)-3*,*4*,*5-trimethoxybenzamide (* ***6*** *)* Yield: 75%; TLC (EtOAc): Rf = 0.80; ^1^H NMR (400 MHz, CDCl_3_): δ 7.01 (*s*, 2H, H(2) and H(6)), 6.40 (*t*, J = 5.3 Hz, 1H, CONH), 3.88 (*s*, 6H, 2 × OCH_3_), 3.87 (*s*, 3H, OCH_3_), 3.45–3.39 (*m*, 4H, NCH_2_(CH_2_)_4_CH_2_Br), 1.90–1.83 (*m*, 2H, CH_2_CH_2_Br), 1.66–1.59 (*m*, 2H, NCH_2_CH_2_), 1.37–1.52 (*m*, 4 H, (CH_2_)_2_(CH_2_)_2_Br); ^13^C NMR (100 MHz, CDCl_3_): δ 167.3 (CONH), 153.2 (C(3) and C(5)), 140.8 (C(4)), 130.3 (C(1)), 104.4 (C(2) and C(6)), 61.0 (OCH_3_), 56.4 (2 × OCH_3_), 40.1 (NCH_2_), 33.9 (CH_2_Br), 32.6 (CH_2_CH_2_Br), 29.6 (NCH_2_ CH_2_), 27.9 (CH_2_(CH_2_)_2_Br), 26.2 (NCH_2_CH_2_ CH_2_); EM/EI m/z (%): 374 (M^+^), 372 (15), 225 (18), 224 (100), 210 (18), 195 (32), 194 (48).

#### General synthetic procedure for obtention of triphenylphosphonium salts (7 and 8)

Bromohexylbenzamide **5**, or **6**, (1 mmol) was mixed with triphenylphosphine (PPh_3_) (1 mmol) in a round-bottomed flask and heated to a temperature of approximately 120 °C for 48 hours. The residue was purified by silica gel flash chromatography using gradient elution (ethyl acetate:methanol from 9:1 to 6:4). The fractions containing the desired compound were collected and the solvent was evaporated to dryness. An amorphous solid was obtained. The reaction was followed by TLC (silica gel, ethyl acetate:methanol (9:1) and dichloromethane:methanol (9:1)). The procedure was adapted from the literature^45^.

*6-(3*,*4-dimethoxybenzamido)hexyltriphenylphosphonium bromide* (**7**) Yield: 65%; TLC (EtOAc:MeOH, 9:1 v/v): Rf = 0.07, (DCM:MeOH, 9:1 v/v): Rf = 0.34; ^1^H NMR (400 MHz, CD_3_OD): δ 7.89–7.73 (*m*, 15 H, PPh_3_), 7.49 (*dd*, J = 8.5 Hz, J = 2.1 Hz, 1H, H(6)), 7.46 (*d*, J = 2.1 Hz, 1H, H(2)), 6.98 (*d*, J = 8.5 Hz, 1H, H(5)), 3.83 (*s*, 6H, 2 × OCH_3_), 3.49–3.42 (*m*, 2H, NCH_2_), 3.37–3.33 (*m*, 2H, CH_2_P^+^Ph_3_), 1.72–1.40 (*m*, 8H, NCH_2_(CH_2_)_4_); ^13^C NMR (100 MHz, CD_3_OD): δ 169.5 (CONH), 153.4 (C(4)), 150.2 (C(3)), 136.3 (*d*, J_CP_ = 3.0 Hz, C(4′)), 134.9 (*d*, J_CP_ = 10.0 Hz, C(2′) and C(6′)), 131.6 (*d*, J_CP_ = 12.6 Hz, C(3′) and C(5′)), 128.1 (C(1)), 122.0 (C(6)), 120.0 (*d*, J_CP_ = 86.2 Hz, C(1′)), 112.2 (C(2)), 112.0 (C(5)), 56.7 (2 × OCH_3_), 40.8 (NCH_2_), 31.2 (*d*, J_CP_ = 16.3 Hz, CH_2_CH_2_P^+^Ph_3_), 30.3 (NCH_2_ CH_2_), 27.2 (CH_2_(CH_2_)_3_P^+^Ph_3_), 23.5 (*d*, J_CP_ = 4.3 Hz, CH_2_(CH_2_)_2_P^+^Ph_3_), 22.7 (*d*, J_CP_ = 51.0 Hz, CH_2_P^+^Ph_3_); EI-MS m/z (%): 511 (M^+^), 277 (37), 263 (40), 262 (100), 183 (87), 165 (47), 151 (35), 108 (44), 107 (29), 77 (26), 52 (26).

*Synthesis of 6-(3*,*4*,*5-trimethoxybenzamido)hexyltriphenylphosphonium bromide (* ***8*** *)* Yield: 79%; TLC (EtOAc:MeOH, 9:1 v/v): Rf = 0.09, (DCM:MeOH, 9:1 v/v): Rf = 0.45; ^1^H NMR (400 MHz, CD_3_OD): δ 7.90–7.75 (*m*, 15H, PPh_3_), 7.28 (*s*, 2H, H(2) and H(6)), 3.95 (*s*, 6H, 2 × OCH_3_), 3.94 (*s*, 3H, OCH_3_), 3.56–3.50 (*m*, 2H, NCH_2_), 3.40–3.37 (*m*, 2H, CH_2_P^+^Ph3), 1.73–1.41 (*m*, 8H, NCH_2_(CH_2_)_4_); ^13^C NMR (100 MHz, CD_3_OD): δ 168.7 (CONH), 154.1 (C(3) and C(5)), 141.6 (C(4)), 136.1 (*d*, J_CP_ = 2.8 Hz, C(4′)), 134.7 (*d*, J_CP_ = 10.0 Hz, C(2′) and C(6′)), 131.4 (*d*, J_CP_ = 12.5 Hz, C(3′) and C(5′)), 130.8 (C(1)), 119.7 (*d*, J_CP_ = 86.1 Hz, C(1′)), 106.0 (C(2) and C(6)), 61.1 (OCH_3_), 57.0 (2 × OCH_3_), 40.6 (NCH_2_), 30.9 (*d*, J_CP_ = 16.2 Hz, CH_2_CH_2_P^+^Ph_3_), 30.0 (NCH_2_ CH_2_), 27.1 (CH_2_(CH_2_)_3_P^+^Ph_3_), 23.3 (*d*, J_CP_ = 4.0 Hz, CH_2_(CH_2_)_2_P^+^Ph_3_), 22.5 (*d*, J_CP_ = 50.8 Hz, CH_2_P^+^Ph_3_); EI-MS m/z (%): 448 (M^+^), 446 (41), 278 (35), 277 (81), 276 (27), 275 (58), 263 (29), 262 (100), 185 (31), 184 (25), 183 (94), 152 (21), 108 (36), 96 (53), 94 (54), 77 (24), 58 (41).

#### General synthetic procedure for obtention of mitochondriotropic antioxidants (AntiOxBEN_1_ and AntiOxBEN_2_)

Triphenylphosphonium salt **7**, or **8**, (1 mmol) was dissolved in 15 mL of anhydrous dichloromethane. The reaction mixture was stirred under argon and cooled at a temperature below −70 °C. Boron tribromide (5–7 mmol, 1 M solution in dichloromethane), was added to the solution and the reaction was kept at −70 °C for 10 minutes. After reaching room temperature, the reaction was continued for 12 hours. Thereafter, the reaction was finished by a slow addition of water (40 mL). After removing the water, the resulting product was dissolved in methanol, dried and the solvent evaporated. The residue was purified by cellulose column chromatography using gradient elution (dichloromethane:methanol from 9:1 to 6:4). The fractions containing the desired compound were collected and the solvent was evaporated to dryness. The resulting residue was crystallized from ethyl ether/methanol to give the corresponding triphenylphosphonium bromide salt as an amorphous solid. The reaction was followed by TLC (silica gel, dichloromethane:methanol (9:1)).The procedure was adapted from the literature^21, 46^.

*6-(3*,*4-dihydroxybenzamido)hexyltriphenylphosphonium bromide* (**AntiOxBEN**_**1**_) Yield: 60%; TLC (DCM:MeOH, 9:1 v/v): Rf = 0.24; ^1^H NMR (400 MHz, DMSO): δ 8.08 (*t*, J = 5.6 Hz, 1H, CONH), 7.94–7.70 (*m*, 15H, PPh_3_), 7.26 (*d*, J = 2.1 Hz, 1H, H(2)), 7.16 (*dd*, J = 8.3 Hz, J = 2.2 Hz, 1H, H(6)), 6.74 (*d*, J = 8.2 Hz, 1H, H(5)), 3.69–3.51 (*m*, 4H, CH_2_P^+^Ph_3_ and 2 × OH), 3.20–3.12 (*m*, 2H, NCH_2_), 1.57–1.39 (*m*, 6H, NCH_2_(CH_2_)_3_), 1.34–1.25 (*m*, 2H, N(CH_2_)_4_CH_2_); ^13^C NMR (100 MHz, DMSO): δ 166.0 (CONH), 148.1 (C(4)), 144.8 (C(3)), 134.9 (*d*, J_CP_ = 2.8 Hz, C(4′)), 133.6 (*d*, J_CP_ = 10.1 Hz, C(2′) and C(6′)), 130.2 (*d*, J_CP_ = 12.4 Hz, C(3′) and C(5′)), 126.0 (C(1)), 118.8 (C(6)), 118.8 (*d*, J_CP_ = 85.6 Hz, C(1′)), 115.1 (C(2)), 114.8 (C(5)), 38.8 (NCH_2_), 29.6 (*d*, J_CP_ = 16.6 Hz, CH_2_CH_2_P^+^Ph_3_), 28.9 (NCH_2_ CH_2_), 25.6 (CH_2_(CH_2_)_3_P^+^Ph_3_), 21.8 (*d*, J_CP_ = 4.2 Hz, CH_2_(CH_2_)_2_P^+^Ph_3_), 20.2 (*d*, J_CP_ = 49.9 Hz, CH_2_P^+^Ph_3_); LRMS/ESI m/z (%): 499 (M^+^ + H- Br, 51), 498 (M^+^-Br, 98), 399 (31), 397 (31), 291 (100), 277 (67). HRMS/ESI calcd for C_31_H_33_NO_3_P^+^(M^+^-Br): 498.2193, found 498.2249.

*6-(3*,*4*,*5-trihydroxybenzamido)hexyltriphenylphosphonium bromide* (**AntiOxBEN**_**2**_) Yield: 50%; TLC (DCM:MeOH, 9:1 v/v): Rf = 0.17; ^1^H NMR (400 MHz, DMSO): δ 8.00 (*t*, J = 5.1 Hz, 1H, CONH), 7.91–7.74 (*m*, 15H, PPh_3_), 6.81 (*s*, 2H, H(2) and H(6)), 3.75–3.39 (*m*, 5H, CH_2_P^+^Ph_3_ and 3 × OH), 3.16–3.11 (*m*, 2H, NCH_2_), 1.50–1.23 (*m*, 8H, NCH_2_(CH_2_)_4_); ^13^C NMR (100 MHz, DMSO): δ 166.3 (CONH), 145.4 (C(3) and C(5)), 136.1 (C(4)), 134.9 (*d*, J_CP_ = 2.7 Hz, C(4′)), 133.6 (*d*, J_CP_ = 10.1 Hz, C(2′) and C(6′)), 130.3 (*d*, J_CP_ = 12.4 Hz, C(3′) and C(5′)), 125.1 (C(1)), 118.6 (*d*, J_CP_ = 85.6 Hz, C(1′)), 106.7 (C(2) and C(6)), 38.9 (NCH_2_), 29.6 (*d*, J_CP_ = 16.6 Hz, CH_2_CH_2_P^+^Ph_3_), 28.9 (NCH_2_ CH_2_), 25.6 (CH_2_(CH_2_)_3_P^+^Ph_3_), 21.8 (*d*, J_CP_ = 4.1 Hz, CH_2_(CH_2_)_2_P^+^Ph_3_), 20.2 (*d*, J_CP_ = 49.8 Hz, CH_2_P^+^Ph_3_); LRMS/ESI m/z (%): 526 (M^+^ + Na- Br, 62), 515 (M^+^ + H- Br, 30), 514 (M^+^-Br, 100), 277 (24). HRMS/ESI calcd for C_31_H_33_NO_4_P^+^(M^+^-Br): 514.2142, found 514.2150.

### Evaluation of AntiOxBENs radical scavenging activity

The radical scavenging activity of AntiOxBENs was evaluated by means of total antioxidant capacity assays based on the spectrophotometric DPPH^•^ and ABTS^•+^ assays.

#### DPPH^•^ radical assay

DPPH^**•**^ radical scavenging activity was performed as previously described^47, 48^. Briefly, solutions with increasing concentrations of the test compounds (range between 50 µM and 500 µM) were prepared in ethanol. A DPPH^**·**^ ethanolic solution (6.85 mM) was also prepared and then diluted to reach the absorbance of 0.72 ± 0.02 at 515 nm. Each compound solution (20 µL) was added to 180 µL of DPPH^**•**^ solution in triplicate, and the absorbance at 515 nm was recorded minutely over 45 minutes. The percent inhibition of the radical was based in the comparison between the blank (20 µL of ethanol and 180 µL of DPPH^**·**^ solution), which corresponded to 100% of radical, and test compounds solutions. The dose-response curves allowed the determination of IC_50_ values.

#### ABTS^•+^ radical cation assay

ABTS^•+^ scavenging activity was evaluated as previously described^49^. Briefly, ethanolic solutions with increasing concentrations of the test compounds (range between 10 µM and 500 µM) were prepared. ABTS^•+^ radical cation solution was obtained by addition of 150 mM aqueous potassium persulfate solution (163 µL) to 10 mL of 7 mM aqueous ABTS solution followed by storage in the dark at room temperature for 16 h (2.45 mM final concentration). The solution was then diluted in ethanol to reach the absorbance of ${\rm{0}}\mathrm{.72}\,\pm \,0{\rm{.02}}$. After addition, in triplicate, of the compound (20 µL) to the ABTS^•+^ solution (180 µL) the spectrophotometric measurement was carried out each minute over a total of 15 minutes. The percent inhibition of radical was based in the comparison between the blank (20 µL of ethanol and 180 µL of ABTS^•+^ solution), which corresponds to 100% of radical, and test compounds solutions. The dose-response curves allowed the determination of IC_50_ values.

### Evaluation of iron chelating properties

The iron chelation capacity of the novel mitochondria-targeted antioxidants was evaluated by the spectrophotometric ferrozine method using a BioTek Synergy HT plate reader, by measuring the absorbance of the [Fe(Ferrozine)_3_]^2+^ complex at 562 nm^50^. The assay was performed in ammonium acetate buffer (pH 6.7) using a solution of ammonium iron (II) sulphate in ammonium acetate as the source of ferrous ions. In each well, a solution of the test compound (100 µM) plus ammonium iron (II) sulphate in ammonium acetate (20 µM) were added, incubated for 10 min and the absorbance was read at 562 nm. An aqueous 5 mM solution of ferrozine was freshly prepared and then added to each well (96 µM final concentration). After a new incubation at 37 °C for a 10 min period, the absorbance of [Fe(ferrozine)_3_]^2+^ complex was measured at 562 nm. Blank wells were ran using DMSO instead of the test compounds. All compounds (protocatechuic and gallic acids, benzoic derivatives, EDTA and MitoQ_10_) as well as ferrozine, were used at a final concentration of 100 µM. The absorbance of the first reading was subtracted to the final values to discard any absorbance due to the test compounds. Data are means ± SEM of three independent experiments and are expressed as % of Fe(II) chelation (EDTA = 100%). EDTA, used as reference, was found to chelate all available iron as it completely inhibited the formation of the coloured ferrozine-fe(II) complex.

### Evaluation of AntiOxCINs redox and lipophilic properties

Electrochemical data were obtained using a computer-controlled potentiostat Autolab PGSTAT302N (Metrohm Autolab, Utrecht, Netherlands). Cyclic voltammetry (CV) was performed at a scan rate of 50 mVs^−1^. The experimental conditions for differential pulse voltammetry (DPV) were: step potential of 4 mV, pulse amplitude of 50 mV and scan rate of 8 mVs^−1^. The electrochemical data were monitored by the General Purpose Electrochemical System (GPES) version 4.9, software package. All electrochemical experiments were performed in an electrochemical cell at room temperature, which was placed in a Faraday cage in order to minimize the contribution of background noise to the analytical signal.

#### Evaluation of redox properties

Voltammetric curves were recorded using a three-electrode system. A glassy carbon electrode (GCE, d = 2 mm) was used as working electrode, the counter electrode was a platinum wire, with a saturated Ag/AgCl reference electrode completing the circuit. Stock solutions of each compound (10 mM) were prepared by dissolving the appropriate amount in ethanol. The voltammetric working solutions were prepared in the electrochemical cell, at a final concentration of 0.1 mM. The supporting electrolyte at pH 7.4 was prepared by diluting 6.2 mL of 0.2 M dipotassium hydrogen phosphate and 43.8 mL of 0.2 M potassium dihydrogen phosphate to 100 mL. Representative voltammograms of AntiOxBEN derivatives are shown in Fig. 4a.

#### Evaluation of lipophilicity

The experimental electrochemical cell used in the evaluation of AntiOxBENs lipophilicity was a four-electrode system with arrays of micro liquid-liquid interfaces (µITIES)^51^. The system contained two Ag/AgCl reference electrodes, prepared by electrochemical oxidation of an Ag wire in NaCl 1 M solution, and two counter electrodes of Pt, one in each phase (Figure S1). The used organic electrolyte salt bis(triphenylphosphoranylidene) ammonium tetrakis(4-chlorophenyl)borate (BTPPATPBCl) was prepared by the metathesis of BTPPACl (97%) and KTPBCl (98%) and 1,6-dichlorohexane (98%) was purified according to a procedure described elsewhere^52^. In this system, a microporous membrane consisting in a 12 µm thick PET membrane with 66 holes, 10 µm hole diameter and 100 µm separation between the holes centres was used. The micro-hole arrays were kindly supplied by Prof. Hubert Girault, Institute of Chemical Sciences and Engineering, ISIC Laboratory of Physical and Analytical Electrochemistry, Switzerland. The electrochemical cell used had a geometrical water/organic solvent interface of 5.2 × 10^−5^ cm^2^. The microporous membrane was sealed with a fluorosilicone sealant (Dow Corning 730) onto a glass cylinder which was filled with 4.0 mL of the aqueous phase, where the aliquots from concentrated AntiOxBEN derivatives solution were added in order to change the concentration of the specie in the aqueous phase. The membrane was then immersed into the organic phase contained in the cell. The organic phase reference solution (a 2 mM BTPPACl + 2 mM NaCl aqueous solution) was mechanically stabilized by a gel^51^. The aqueous supporting electrolyte solution used in the studies was a Tris-HCl buffer 10 mM pH 7.0. An example of representative data is depicted in Fig. 4b.

### Evaluation of AntiOxBENs functional mitochondrial profile

#### Animal handling and isolation of liver mitochondria

Animals: Male Wistar-Han rats (10 weeks old) were housed in our accredited animal colony (Laboratory Research Center, Faculty of Medicine of University of Coimbra). Animals were group-housed in type III-H cages (Tecniplast, Italy) and maintained in specific environmental requirements (22 °C, 45–65% humidity, 15–20 changes/hour ventilation, 12 h artificial light/dark cycle, noise level <55 dB) and with free access to standard rodent food (4RF21 GLP certificate, Mucedola, Italy) and acidified water (at pH 2.6 with HCl to avoid bacterial contamination). This research procedure was carried out in accordance with European Requirements for Vertebrate Animal Research and approved by the animal welfare commission of the Center for Neuroscience and Cell Biology, University of Coimbra, Portugal. Further approval was obtained from the National Agency for Veterinary and Agriculture (DGAV), reference 0421/000/000/2016.

Rat liver mitochondria (RLM) were prepared by tissue homogenization followed by differential centrifugations in ice-cold buffer containing 250 mM sucrose, 10 mM HEPES (pH 7.4), 1 mM EGTA, and 0.1% fat-free bovine serum albumin. After obtaining a crude mitochondrial preparation, pellets were washed twice and resuspended in washing buffer (250 mM sucrose and 10 mM HEPES, pH 7.4)^53^. The protein concentration was determined by the biuret assay using bovine serum albumin (BSA) as a standard^54^.

### AntiOxBENs mitochondrial uptake

The uptake of AntiOxBEN derivatives by energized RLM was evaluated by using an ion-selective electrode, according to previously established methods, which measures the distribution of tetraphenylphosphonium (TPP^+^)^29^. An Ag/AgCl electrode was used as reference. To measure AntiOxBENs uptake, RLM (0.5 mg protein/mL) were incubated under constant stirring, at 37 °C, in 1 mL of KCl medium (120 mM KCl, 10 mM HEPES, pH 7.2 and 1 mM EGTA). Five sequential 1 μM additions of AntiOxBEN derivatives were performed to calibrate the electrode response in the presence of rotenone (1.5 μM). Succinate (SUC, 10 mM) was then added to generate ΔΨ, while valinomycin (VAL, 0.2 μg/mL) was added at the end of the assay to dissipate ΔΨ. The mitochondrial accumulation ratio was calculated by the disappearance of AntiOxBEN derivatives from extra- to intramitochondrial medium assuming an intramitochondrial volume of ∼0.5 μL/mg protein and a binding correction expected for the mitochondrial uptake of TPP compounds.

### Evaluation of AntiOxBENs effect on RLM lipid peroxidation

The effects of AntiOxBENs on RLM lipid peroxidation were evaluated by two distinct methods.

(A) RLM lipid peroxidation was measured by oxygen consumption as described by Sassa *et al*.^22^. The oxygen consumption of 2 mg RLM in a total volume of 1 mL of a reaction medium consisting of 100 mM KCl, 10 mM Tris-HCl and pH 7.6, using glutamate/malate (5 mM/2.5 mM) as respiratory substrate, was monitored at 37 °C with a Clark-type oxygen electrode. RLM were incubated for a 5 min period with the different compounds (5 µM) and the lipid peroxidation process started by adding 10 mM ADP and 0.1 mM FeSO_4_ (final concentrations). The saturated concentration of O_2_ in the incubation medium was assumed to be 217 µM at 37 °C. Time-dependent changes on oxygen consumption resulting from peroxidation of RLM membranes by a pro-oxidant pair (1 mM ADP/0.1 mM FeSO_4_) were recorded (Figure S2). The time lag-phase associated with the slower oxygen consumption that followed the addition of ADP/Fe^2+^ was used to measure the effectiveness of the tested compounds to prevent lipid peroxidation.
(B) Lipid peroxidation was also measured by thiobarbituric acid reactive species (TBARS) assay^21^. RLM (2 mg protein/ml) were incubated in 0.8 mL medium containing 100 mM KCl, 10 mM Tris-HCl and pH 7.6, at 37 °C, supplemented with 5 mM glutamate/2.5 mM malate as substrate. RLM were incubated for a 5 min period with the different tested compounds (5 µM) and then mitochondria were exposed to oxidative stress condition by the addition of 100 µM FeSO_4_/500 µM H_2_O_2_/5 mM ascorbate for 15 min at 37 °C. After exposure to oxidative stress, 60 µL of 2% (v/v) butylated hydroxytoluene in DMSO was added, followed by 200 µL of 35% (v/v) perchloric acid and 200 µL of 1% (w/v) thiobarbituric acid. Samples were then incubated for 15 min at 100 °C, allowed to cool down and the supernatant transferred to a glass tube. After addition of 2 mL MiliQ water and 2 mL butan-1-ol, samples were vigorously vortexed for few seconds and the two phases were allowed to separate. The fluorescence of aliquots (250 µL) of the organic layer was analyzed in a plate reader (λ_Ex_ = 515 nm; λ_Em_ = 553 nm) for TBARS. The TBARS background production in RLM energized with glutamate/malate was found to be negligible.

### Evaluation of AntiOxBENs effect on mitochondrial respiration

RLM respiration was evaluated polarographically with a Clark-type oxygen electrode, connected to a suitable recorder in a 1 mL thermostated water-jacketed chamber with magnetic stirring, at 37 °C^55^. The standard respiratory medium consisted of 130 mM sucrose, 50 mM KCl, 5 mM KH_2_PO_4_, 5 mM HEPES (pH 7.3) and 10 μM EGTA. Increasing concentrations of AntiOxBEN derivatives (2.5–10 μM) were added to the reaction medium containing respiratory substrates glutamate/malate (10 mM and 5 mM respectively) or succinate (5 mM) and RLM (1 mg) and allowed to incubate for a 5 min period prior to the assay. State 2 was considered as the respiration during the 5 min incubation time with AntiOxBENs. To induce state 3 respiration, 125 nmol ADP (using glutamate/malate) or 75 nmol ADP (using succinate) was added. State 4 was determined after ADP phosphorylation finished. Subsequent addition of oligomycin (2 μg/ml) inhibited ATP-synthase and originated the oligomycin-inhibition respiration state. Finally, 1 μM FCCP was added to uncouple respiration.

### Evaluation of AntiOxBENs effect on mitochondrial transmembrane electric potential (ΔΨ)

Mitochondrial transmembrane electric potential (ΔΨ) was estimated through the evaluation of fluorescence changes of the dye safranine (5 μM), as recorded on a spectrofluorometer operating at excitation and emission wavelengths of 495 and 586 nm, with a slit width of 5 nm^56^. Increasing concentrations of AntiOxBENs (2.5–10 μM) were added to the reaction medium (200 mM sucrose, 1 mM KH_2_PO_4_, 10 mM Tris at pH 7.4 and 10 μM EGTA) containing respiratory substrates glutamate/malate (5 mM and 2.5 mM respectively) or succinate (5 mM) and RLM (0.5 mg in 2 mL final volume) and allowed to incubate for a 5 min period prior to recording, at 25 °C. In this assay, maximum ΔΨ was measured after the addition of safranine (5 μM) and ADP (25 nmol) were used to initiate the assay and to induce depolarization, respectively. Moreover, 1 μM FCCP was added at the end of all experiments to induce uncoupled respiration. Repolarization corresponded to the recovery of ΔΨ, after the complete phosphorylation of the added ADP. Lag phase reflected the time required to phosphorylate the added ADP. ΔΨ was calculated using a calibration curve obtained when RLM were incubated in a K^+^-free reaction medium containing 200 mM sucrose, 1 mM NaH_2_PO_4_, 10 mM Tris (pH 7.4) and 10 μM EGTA, supplemented with 0.4 μg valinomicin. The extension of safranine fluorescence changes of resulting from ΔΨ fluctuations were similar in the standard and K^+^-free medium. Isolated RLM developed a ΔΨ ≈ 230 mV and ΔΨ ≈ 186 mV (negative inside) when glutamate/malate or succinate were used as substrates, respectively.

### Evaluation of AntiOxBENs cytotoxicity

#### Cell lines and culture conditions

Rat embryonic myocardium (H9c2) (ATCC, VA, USA), human hepatocellular carcinoma cells (HepG2) (ECACC, UK) and human neonatal dermal fibroblasts (HNDF) (ATCC, Barcelona, Spain) were used in this study. Cells were cultured in low-glucose medium (5 mM) composed by Dulbecco’s modified Eagle’s medium (DMEM; D5030) supplemented with sodium pyruvate (0.11 g/L), sodium bicarbonate (3.7 g/L), HEPES (1.19 g/L), 6 mM glutamine) and 10% fetal bovine serum (FBS) and 1% of antibiotic penicillin-streptomycin 100x solution. All cells were cultured under 5% CO_2_ atmosphere at 37 °C and passaged by tripsinization when reaching 70–80% confluence.

### Evaluation of AntiOxBENs effects on metabolic activity in the presence and absence of oxidative stressors

To evaluate the cytotoxic effects of AntiOxBENs on different cell lines, resazurin reduction to resorufin by dehydrogenases present in viable cells, thus indirectly measuring metabolic viability, was used as end-point. H9c2 (7.5 × 10^3^ cells/well), HepG2 (2.5 × 10^4^ cells/mL) and HNDF (7.5 × 10^3^ cells/mL) cells were seeded in a 96-well plate and proliferate for 24 hours before treatment. Increasing concentrations of AntiOxBENs were then added to cells for 48 hours. After incubation time, cellular metabolic activity and total mass was determined as previously described^57^. Briefly, the medium was replaced by fresh medium containing resazurin (10 µg/mL) prepared in sterile PBS (1X) and left to react for 1 hour. The fluorescent signal was monitored using a 540 nm excitation wavelength and 590 nm emission wavelength in a Cytation 3 reader (BioTek Instruments Inc., USA). Results are means ± SEM of four independent experiments. The AntiOxBENs antioxidant efficiency in the presence of oxidative stressors was also evaluated. H9c2 (7.5 × 10^3^ cells/well), HepG2 (2.5 × 10^4^ cells/well) and HNDF (7.5 × 10^3^ cells/well) cells were seeded in a 96-well plate and allowed to proliferate for 24 hours before treatment. Initially, three different concentrations of AntiOxBENs (25, 50 and 100 µM) were added to cells for 24 hours and then the oxidative stress-induced agent t-BHP (150 µM, 500 and 250 µM) was added to H9c2, HepG2 and HNDF cells for 3, 24 and 3 hours more, respectively. After incubation time, cellular metabolic activity and total mass was determined as previously described.

### Evaluation of AntiOxBENs effects on ATP levels

Intracellular ATP levels was measured by using CellTiter-Glo® Luminescent Cell Viability Assay (Promega) following manufacturer’s instructions. H9c2 (7.5 × 10^3^ cells/well), HepG2 (2.5 × 10^4^ cells/well) and HNDF (7.5 × 10^3^ cells/well) cells were seeded in 100 µL of culture medium, in a white opaque-bottom, 96-well plate and proliferate for 24 hours before treatment. Increasing concentrations of AntiOxBENs were then added to cells for 48 hours. After incubation time, 50 µL of culture medium was removed from the wells and 50 µL of medium containing CellTiter-Glo® Reagent (CellTiter-Glo® Buffer + CellTiter-Glo® Substrate) was added to the cells. Contents were mixed for 2 minutes on an orbital shaker to induce cell lysis and, after 10 minutes of incubation at 22 °C, the luminescence signal was monitored in a Cytation 3 reader (BioTek Instruments Inc., USA). ATP standard curve was also generated following manufacturer’s instructions. Luminescence signal is proportional to the amount of ATP present in solution.

### Statistics

Data were analyzed in GraphPad Prism 5.0 software (GraphPad Software, Inc.), with all results being expressed as means ± SEM for the number of experiments indicated. The student’s t-test for comparison of two means, and one-way ANOVA with Dunnet multiple comparison post-test was used to compare more than two groups with one independent variable were used in data analysis. Significance was accepted with *P < 0.05, **P < 0.01, ***P < 0.0005, ****P < 0.0001.

### Data availability

The datasets generated during and/or analysed during the current study are available from the corresponding author on reasonable request.

## Electronic supplementary material

Supplementary Information
