# Supplementary material for: Development of hydroxybenzoic-based platforms as a solution to deliver dietary antioxidants to mitochondria
Source: Sci Rep. 2017 Jul 28;7:6842. doi: 10.1038/s41598-017-07272-y (PMC5533782; doi:10.1038/s41598-017-07272-y)
Supplement: Supplementary file 1 — Supplementary Information [file 41598_2017_7272_MOESM1_ESM.pdf]

SREP-17-07209-R1

Development of hydroxybenzoic-based platforms as a solution to deliver dietary antioxidants to mitochondria

José Teixeira[a,b¥], Catarina Oliveira[a¥], Ricardo Amorim[a,b], Fernando Cagide[a], Jorge Garrido[a,c], José A. Ribeiro[a], Carlos M. Pereira[a], António F. Silva[a], Paula B. Andrade[d], Paulo J. Oliveira[b*], Fernanda Borges[a*]

[a]*CIQUP/Department of Chemistry and Biochemistry, Faculty of Sciences, University of Porto, Porto 4169-007, Portugal*

[b] *CNC – Center for Neuroscience and Cell Biology, UC-Biotech Building, Biocant Park –University of Coimbra, Cantanhede 3060-197, Portugal*

[c] *Department of Chemical Engineering, School of Engineering (ISEP), Polytechnic of Porto, Porto 4200-072, Portugal*

[d] REQUIMTE/LAQV-Laboratory of Pharmacognosy, Department of Chemistry, Faculty of Pharmacy, University of Porto, Porto 4050-313, Portugal

Corresponding Authors

Fernanda Borges, CIQ/Department of Chemistry and Biochemistry, Faculty of Sciences, University of Porto, Porto 4169-007, Portugal, Porto 4169-007, Portugal.

E-mail: fborges@fc.up.pt

Paulo J. Oliveira, CNC – Center for Neuroscience and Cell Biology, University of Coimbra, UC-Biotech, Biocant Park 3060-197 Cantanhede, Portugal.

E-mail: pauloliv@cnc.uc.pt

Supporting Information

S1. Toxicity effects of MitoQ10 on liver mitochondrial respiration and on ΔΨ

The toxicity profile of the new mitochondria-targeted antioxidants was assessed on isolated RLM fractions by the evaluation of their direct effects on the bioenergetics apparatus.

The first studies on mitochondrial respiration were performed with the mitochondrial-targeted antioxidant MitoQ10 not only as a standard, but also used to set the range of concentration used. The highest concentration used was the one at which MitoQ10 completely disrupted mitochondrial bioenergetics. For all tested concentrations, MitoQ10 caused a significant decrease of RCR and ADP/O parameters (**Supplementary Table S1**). Moreover, when RLM were incubated with MitoQ10 in concentrations up to 2.5 μM, an increase on state 2, state 4 and oligomycin-inhibited respiration and a decrease on state 3 and FCCP-uncoupled respiration was observed, using glutamate/malate as substrate. When using succinate as substrate, RLM were completely uncoupled in the presence of MitoQ10 at the highest concentration tested (5 μM) (**Supplementary Figure S4**). The incubation with increasing concentrations of MitoQ10 resulted in a progressive decrease of the maximum ΔΨ obtained upon energization. ΔΨ collapse after ADP addition was observed with 10 μM MitoQ10, since no repolarization occurred after ADP-induced depolarization (**Supplementary Table S1**).

S2. Supplementary Figures

Ag	AgCl	NaCl 2 mM<br>BTPPACl 2 mM<br>*(Water)*	BTPPATPBCl<br>1 mM<br>*(DCH)*	*x* mM **AntiOxBEN$^+$**<br>Tris-HCl 10 mM pH 7.0<br>*(Water)*	AgCl	Ag’

Supplementary Figure S1 - Evaluation of AntiOxBENs lipophilicity in water/DCH. Schematic representation of the electrochemical cell used in the AntiOxBENs transfer across the aqueous phase/DCH micro-interface at pH 7.0.

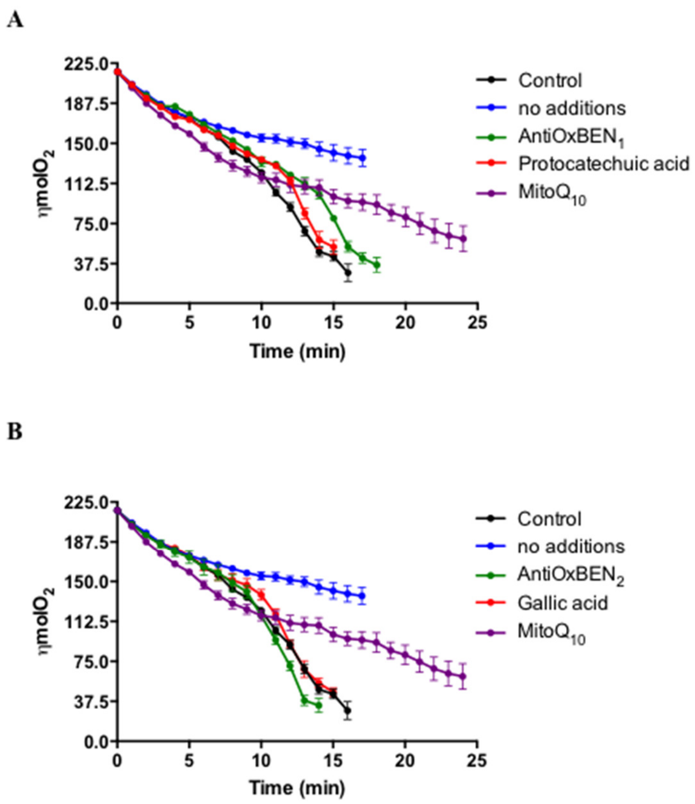

Supplementary Figure S2 - Typical recording of the effect of benzoic acid and AntiOxBEN derivatives, containing a (A) catechol (protocatechuic acid and AntiOxBEN$_1$) or (B) pyrogallol (gallic acid and AntiOxBEN$_2$) core on lipid peroxidation of RLM membranes induced by ADP and Fe2+ followed by oxygen consumption. MitoQ$_{10}$, the golden standard for a mitochondria-targeted antioxidants, was used as control for all mitochondriotropic compounds. The traces are means ± SEM recording from six independent experiments.

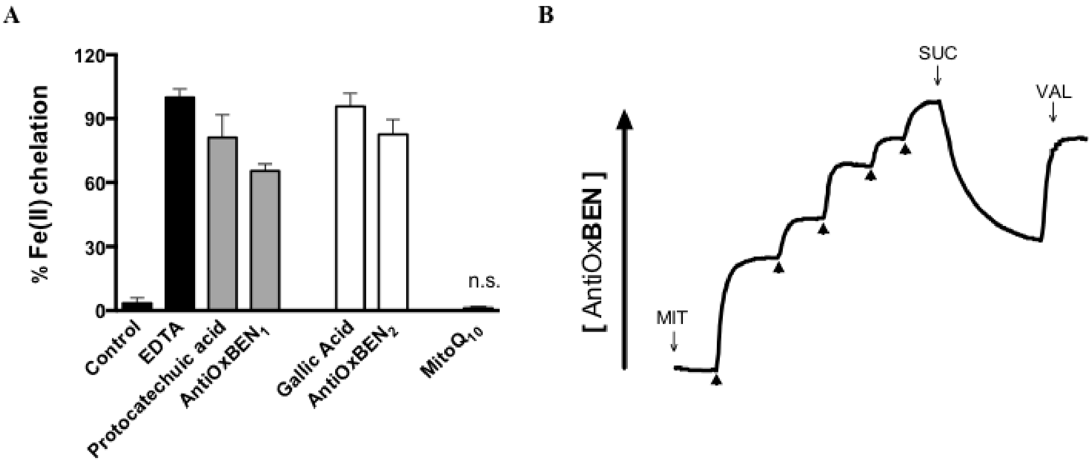

C

	[Intramitochondrial] (mM)	[Extramitochondrial] (µM)	Accumulation Ratio
**$AntiOxBEN_1$**	1.5	1.1	2200
**$AntiOxBEN_2$**	1.8	2.1	1900

Supplementary Figure S3 - (A) Evaluation of iron chelating properties of AntiOxBENs and $MitoQ_{10}$. EDTA (chelating agent) was used as reference (EDTA = 100%). Data are means ± SEM of three independent experiments and are expressed as % of control (EDTA = 100%). Statistically significant compared with control group using one-way ANOVA ($P<0.0001$, n.s., not significant). (B) AntiOxBENs uptake by energised rat liver mitochondria measured by using a TPP-selective electrode. (C) AntiOxBENs accumulation ratio by rat liver mitochondria.

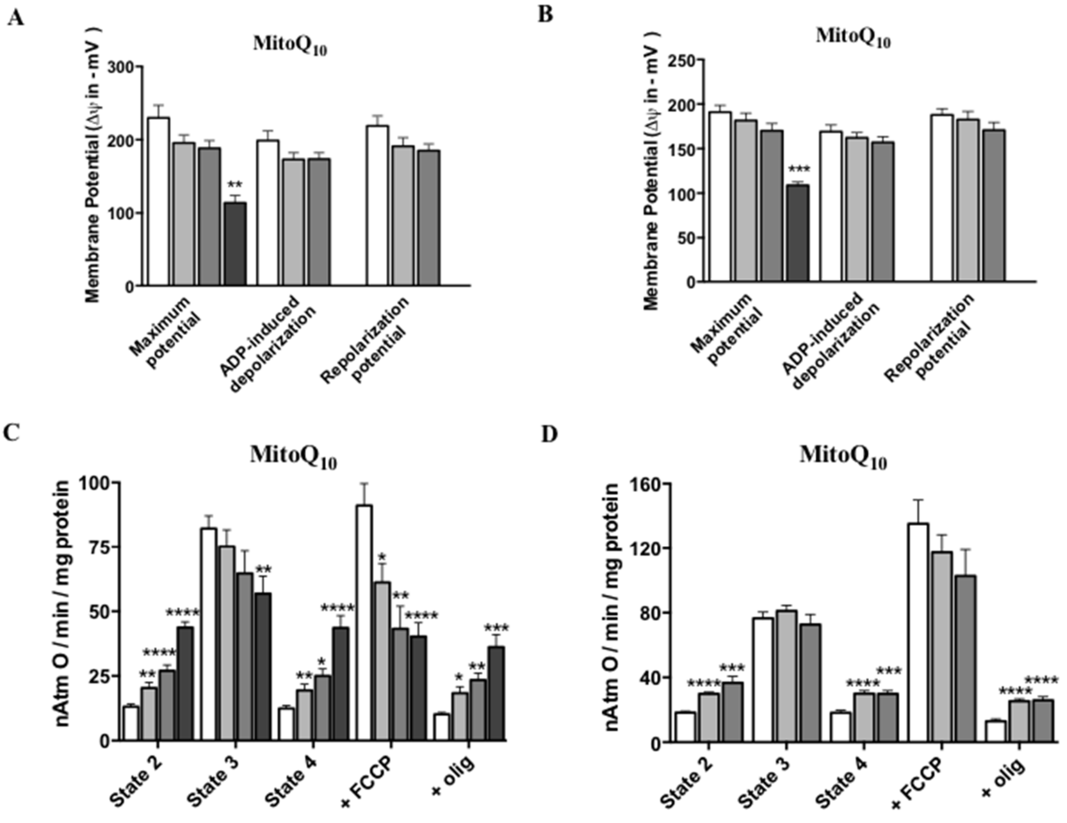

Supplementary Figure S4 – Effects of $MitoQ_{10}$ on RLM (A/B) transmembrane electric potential (ΔΨ) and (C/D) respiration supported by 10 mM glutamate + 5 mM malate (A/C) or 5 mM succinate (B/D). The white bars refer to the control, while grey bars refer to the experiments where RLM were pre-incubated with $MitoQ_{10}$ (2.5µM – light grey; 5µM – grey; and 10 µM – dark grey). The presented results are means ± SEM of seven independent experiments. The statistical significance relative to the different bioenergetics parameters was determined using Student's two tailed t-test (*P<0.05, **P<0.01, ***P<0.0005, ****P<0.0001).

S2. Supplementary Tables

Supplementary Table S1. Effect of $MitoQ_{10}$ on mitochondrial bioenergetics: mitochondrial respiratory control ratio (RCR) and efficiency of the phosphorylative system (ADP/O).

**Mitochondrial Bioenergetics**		Control	$MitoQ_{10}$		
			**2.5 µM**	**5 µM**	**10 µM**
**Glutamate/Malate**	**RCR**	7.3 ± 0.6	4.2 ± 0.6 **	2.7 ± 0.3 ****	1.3 ± 0.1 ****
	**ADP/O**	2.6 ± 0.1	2.2 ± 0.1 *	1.9 ± 0.1 ****	2.0 ± 0.2 ***
**Succinate**	**RCR**	4.1 ± 0.3	2.6 ± 0.2 **	2.4 ± 0.2 ***	
	**ADP/O**	1.5 ± 0.1	1.3 ± 0.1 *	1.3 ± 0.1 *	

Effect of $MitoQ_{10}$ on RCR and ADP/O of energized mitochondria (5 mM glutamate / 2.5 malate or 5 mM succinate). Values are means ± SEM of seven independent experiments. Statistically significant compared with control using Student's two tailed t-test (*P<0.05, **P<0.01, ***P<0.0005, ****P<0.0001).

S3. NMR spectra of the mitochondriotropic antioxidants (AntiOxBEN$_1$ and AntiOxBEN$_2$)

AntiOxBEN$_1$

^{1}H NMR

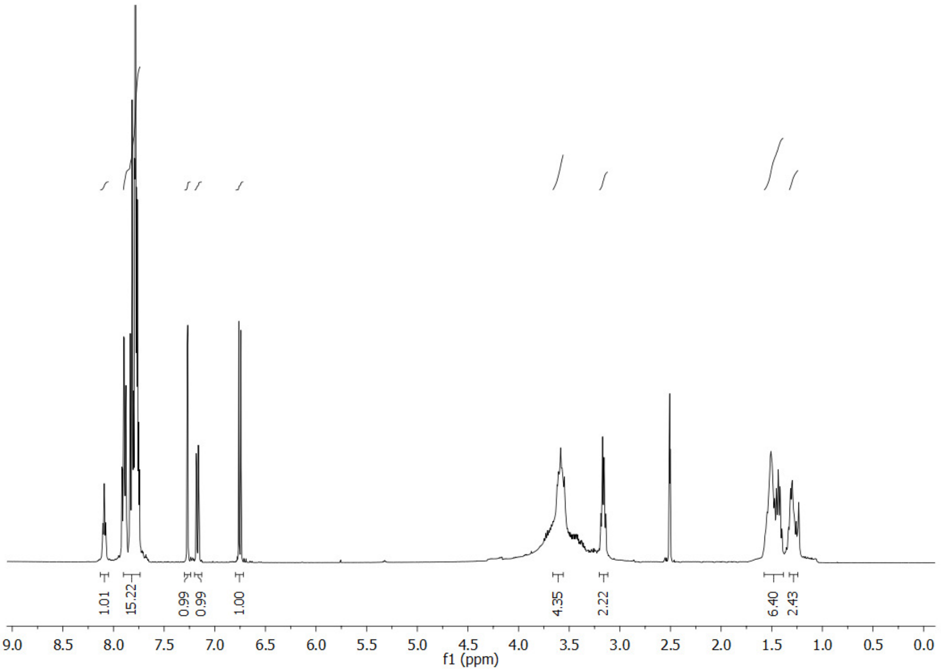

^{13}C NMR

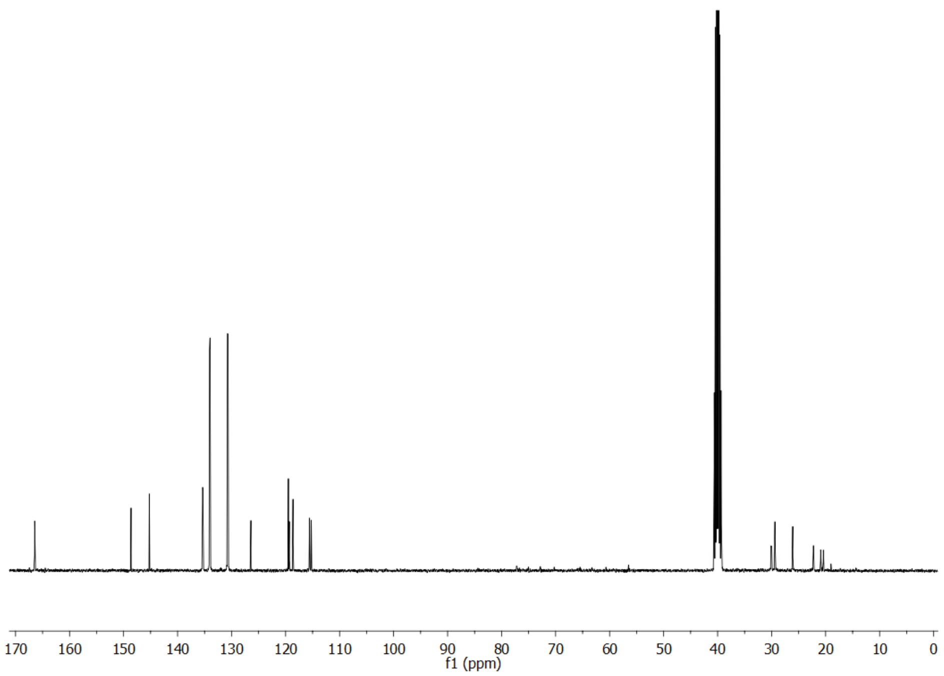

DEPT 135

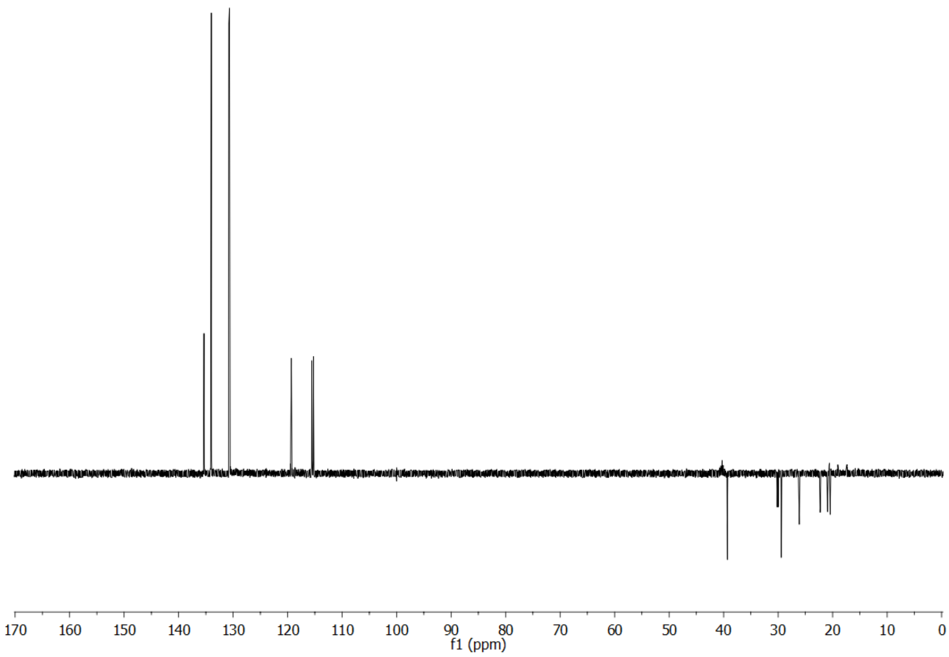

AntiOxBEN$_2$

^{1}H NMR

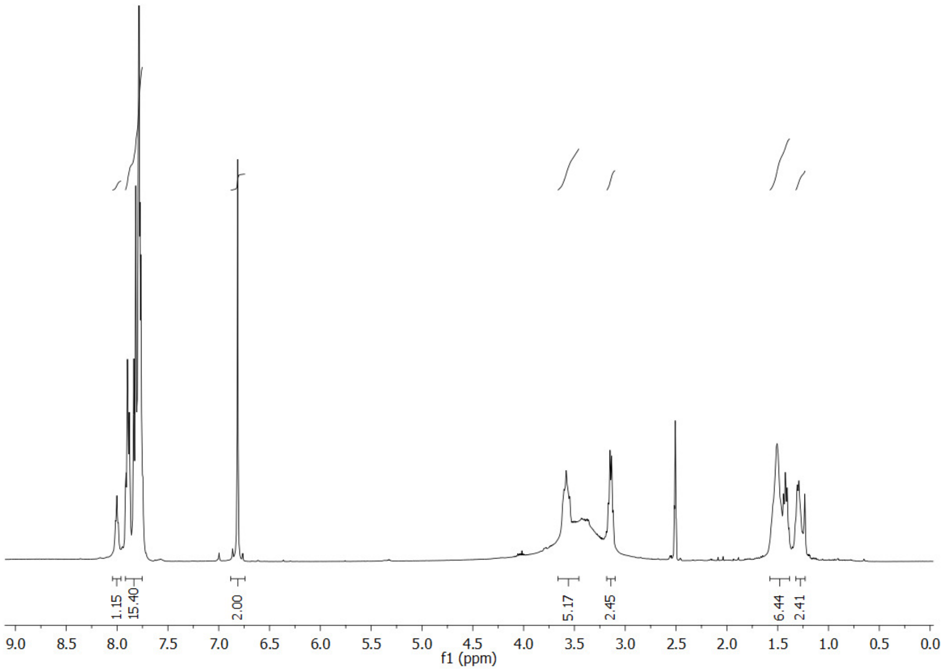

^{13}C NMR

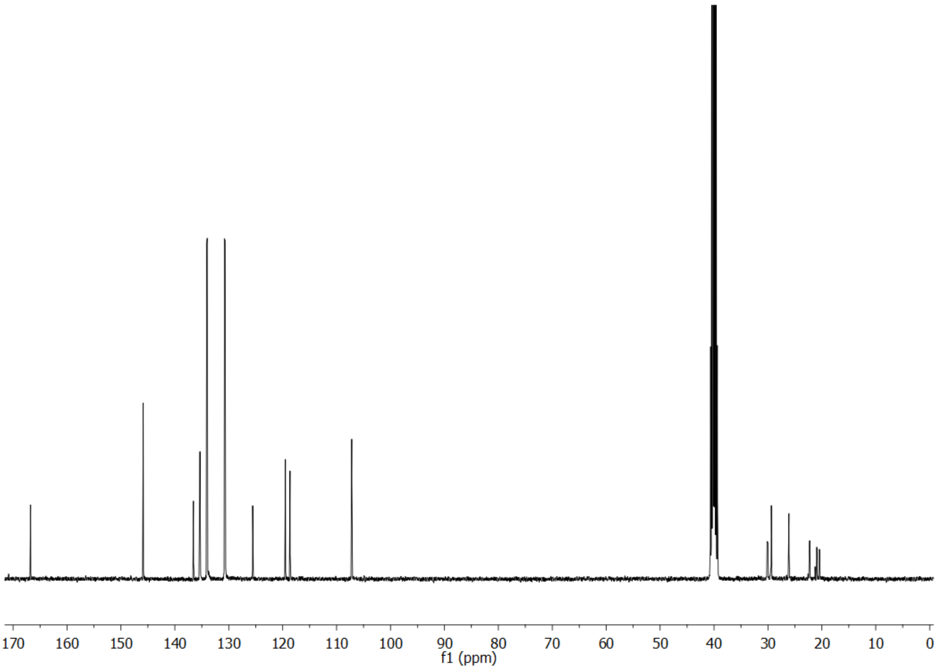

DEPT 135

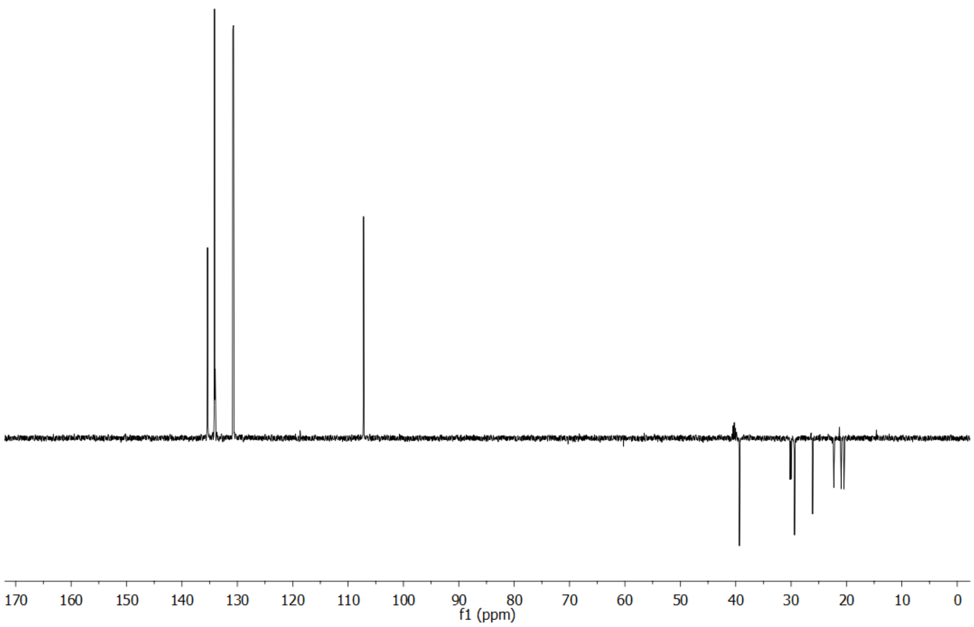
170 160 150 140 130 120 110 100 90 80 70 60 50 40 30 20 10 0
f1 (ppm)
